# High Efficiency Gene Correction in Hematopoietic Cells by Donor-Template-Free CRISPR/Cas9 Genome Editing

**DOI:** 10.1016/j.omtn.2017.11.001

**Published:** 2017-11-10

**Authors:** Duran Sürün, Joachim Schwäble, Ana Tomasovic, Roy Ehling, Stefan Stein, Nina Kurrle, Harald von Melchner, Frank Schnütgen

**Affiliations:** 1 Department of Molecular Hematology, Goethe University Medical School, 60590 Frankfurt am Main, Germany; 2 LOEWE Center for Cell and Gene Therapy, Goethe University Medical School, 60590 Frankfurt am Main, Germany; 3 Institute for Transfusion Medicine und Immunohematology, Goethe University Medical School, 60528 Frankfurt am, Germany; 4 Institute for Tumor Biology and Experimental Therapy, Georg-Speyer-Haus, 60596 Frankfurt am Main, Germany

**Keywords:** CRISPR/Cas9, non-homologous end joining, NHEJ, *in situ* gene correction, hematopoietic cells, chronic granulomatous disease, CGD

## Abstract

The CRISPR/Cas9 prokaryotic adaptive immune system and its swift repurposing for genome editing enables modification of any prespecified genomic sequence with unprecedented accuracy and efficiency, including targeted gene repair. We used the CRISPR/Cas9 system for targeted repair of patient-specific point mutations in the Cytochrome b-245 heavy chain gene (*CYBB*), whose inactivation causes chronic granulomatous disease (XCGD)—a life-threatening immunodeficiency disorder characterized by the inability of neutrophils and macrophages to produce microbicidal reactive oxygen species (ROS). We show that frameshift mutations can be effectively repaired in hematopoietic cells by non-integrating lentiviral vectors carrying RNA-guided Cas9 endonucleases (RGNs). Because about 25% of most inherited blood disorders are caused by frameshift mutations, our results suggest that up to a quarter of all patients suffering from monogenic blood disorders could benefit from gene therapy employing personalized, donor template-free RGNs.

## Graphical Abstract

**Figure:**
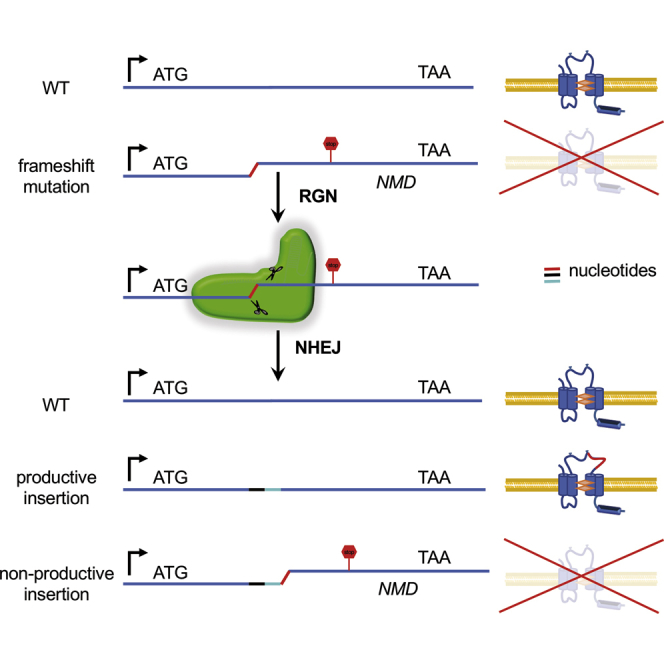

## Introduction

The vast majority of inherited monogenic disorders are caused by patient-specific mutations dispersed over the entire locus of the affected gene.^1^ Although correcting mutations by introducing healthy gene copies into the genome of diseased cells proved effective in several clinical gene therapy trials,^2^ insertional mutagenesis and unregulated transgene expression remain a concern for randomly integrating vectors (reviewed by Naldini^3^).

Ideally, diseased genes would be corrected directly at their endogenous loci by homologous recombination (HR). Although the original technology developed for gene targeting in mouse embryonic stem cells was successfully upscaled for high throughput generation of knockout mice,^4^ its efficiency is quite variable and ineffective in human somatic cells. This changed considerably with the development of designer endonucleases capable of inducing DNA double-strand breaks (DSBs) in any pre-specified genomic sequence that are restored either by homology directed repair (HDR) or non-homologous end joining (NHEJ). Whereas HDR uses a donor DNA template and can be exploited to create specific sequence changes, including targeted addition of whole genes, NHEJ repairs DSBs in the absence of a donor template by religating DNA ends—an error prone process associated with random nucleotide insertions or deletions (indels).

Successful correction of human disease mutations in hematopoietic and induced pluripotent stem cells by designer endonucleases has thus far been based exclusively on HDR. Although HDR offers precision, efficiency is low and most editing protocols rely on positive selection to enrich for gene-corrected cells.^5^, ^6^, ^7^, ^8^, ^9^, ^10^, ^11^, ^12^ Because DSB repair by NHEJ in mammalian cells significantly exceeds HDR and, more importantly, is the dominant DSB-repair pathway in hematopoietic stem and progenitor cells (HSPCs),^13^, ^14^ we exploited NHEJ for gene repair because, in theory, approximately one-third of indels associated with NHEJ should restore the open reading frame (ORF) disrupted by a disease mutation. This could lead to many ORF reconstitutions, of which some, depending on the position and type of the original mutation, should completely or partially recover protein function, as has been shown recently for the dystrophin gene in patients with Duchenne’s muscular dystrophy (DMD).^15^

Here, we show that gene-inactivating point mutations introduced into EGFP transgenes expressed in PLB-985 myeloid leukemia cells are effectively repaired by donor template-free RNA-guided CRISPR/Cas9 endonucleases (RGNs) delivered by integrase-defective lentiviruses (IDLVs). Additionally, mutations in the Cytochrome b-245 heavy chain (*CYBB*) gene encoding nicotinamide adenine dinucleotide phosphate (NADPH) oxidase catalytic gp91^phox^ subunit causing X-linked chronic granulomatous disease (XCGD; a life-threatening primary immunodeficiency disorder^16^) can be functionally reconstituted in *CYBB*-null PLB (XCGD) cells^17^ engineered to express patient-specific *CYBB* mutations. With gene repair efficiency of up to 25% for some *CYBB* mutations and an on-target mutation rate of 75% at the endogenous *CYBB* locus, we believe that a donor template-free RGN approach has potential for personalized gene therapy of chronic granulomatous disease (CGD) and other monogenic blood disorders.

## Results and Discussion

**Figure 1:**
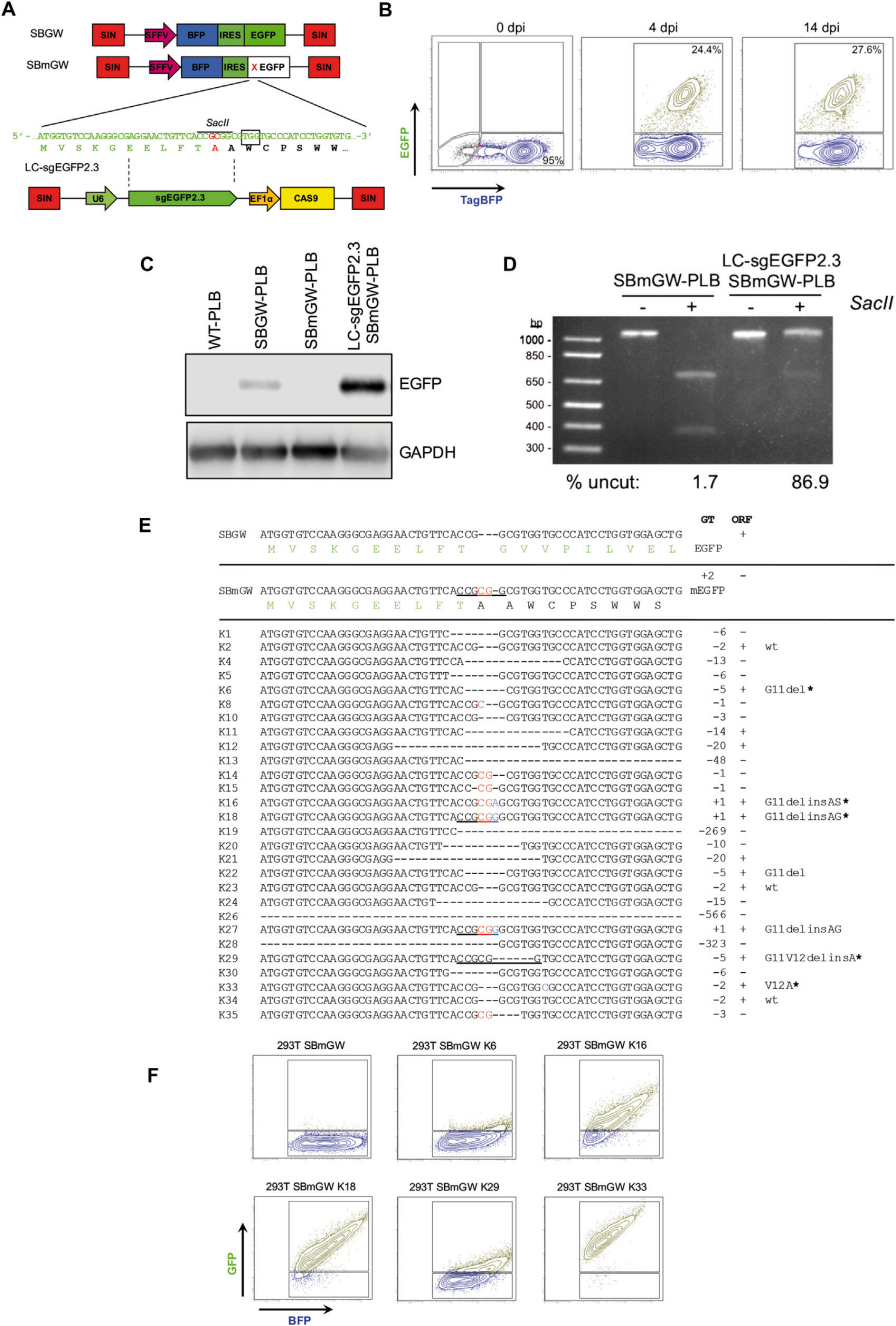
EGFP Repair Efficiency in PLB Cells Expressing Dual Color Reporters (A) Lentiviral reporter constructs with cDNAs encoding blue fluorescent protein (tag BFP) and either wild-type (SBGW) or mutated (SBmGW) EGFP (top) and schematic representation of the LC-sgEGFP2.3 lentiviral vector with its target sequence (bottom). (B) Frequency of EGFP^+^ cells among FACS-sorted BFP^+^ SBmGW PLB cells before and after LC-sgEGFP2.3 IDLV infection (MOI 11). (C) Western blot showing EGFP expression in WT, unsorted SBGW-PBL control cells and in sorted BFP^+^ SBmGW-PLB cells before and after IDLV infection. (D) *SacII* digests of genomic EGFP amplification products from SBmGW-PLB cells before and after IDLV treatment. Numbers at the bottom represent the amount of uncut DNA estimated by densitometry. (E) Indel sequences recovered by shot-gun cloning. Reconstituted *SacII* restriction sites are underlined. (F) FACS analysis of HEK293T cells expressing mEGFP cDNAs reconstituted by non-canonical ORFs. For further explanation, see text. BFP, blue fluorescent protein; EF1a, elongation factor 1 alpha short variant promoter; EGFP, enhanced green fluorescent protein; IRES, internal ribosomal entry site; pA, polyadenylation site; SFFV, spleen focus forming virus promoter; SIN, self-inactivating long terminal repeat (LTR); U6, human RNA polymerase III promoter.

To test gene repair efficiency by NHEJ in human hematopoietic cells, we generated PLB-985 (PLB)^18^ reporter cells expressing blue fluorescent protein (tagBFP),^19^, ^20^ along with either intact (EGFP) or mutationally inactivated EGFP (mEGFP). TagBFP (BFP) was linked to EGFP or mEGFP by an internal ribosomal entry site (IRES), and BFP-IRES-EGFP cassettes were cloned into a self-inactivating (SIN) lentiviral vector downstream of an internal SFFV promoter (Figure 1A). The EGFP mutation consisted of a 2-nt, frameshifting insertion that generated a *SacII* restriction site at the 5′ end of EGFP (Figure 1A). Two lentiviral vectors, SBGW and SBmGW, were used to infect PLB cells (PLBs) at a low multiplicity (MOI 0.01) to obtain single copy integrations (Figure S1). Two days after infection, transduced PLBs were analyzed by fluorescence-activated cell sorting (FACS). As expected, the majority of SBGW-transduced PLBs (SBGW-PLB) were double positive for BFP and EGFP (BFP^+^GFP^+^), whereas, consistent with EGFP inactivation, SBmGW-transduced PLBs (SBmGW-PLB) expressed only BFP (Figure S2).

Next, we cloned a single guide RNA (sgRNA) targeting the EGFP mutation (sgEGFP2.3, Figure 1A) into the pLentiCRISPRv2 lentiviral vector^21^ and infected FACS-sorted BFP^+^SBmGW-PLBs with IDLVs referred to as LC-sgEGFP2.3. IDLVs were chosen for transient RGN delivery because they can effectively transduce hematopoietic stem cells.^6^, ^22^, ^23^ More recently, however, direct RGN delivery by electroporation deemed clinically more compatible was shown to be at least as effective as IDLVs.^12^, ^24^ Figure 1B shows infection of BFP^+^SBmGW-PLBs with LC-sgEGFP2.3 reconstituted EGFP expression in up to 24% of cells within 4 days post-infection. After 14 days, the fraction of BFP^+^EGFP^+^ cells increased by another 3%, indicating stable EGFP repair (Figure 1B). Furthermore, western blot analysis revealed robust EGFP protein expression (Figure 1C).

To estimate the on-target mutation rate of LC-sgEGFP2.3, we digested genomic EGFP amplification products from the transduced SBmGW-PLBs with *SacII*, separated restriction fragments on agarose gels, and quantified uncleaved DNA by densitometry. Figure 1D shows that up to 87% of EGFP alleles lost the *SacII* restriction site, which is consistent with a high IDLV-transduction rate.

To determine the type of indels leading to EGFP repair, we shot-gun cloned genomic EGFP amplification products from IDLV-transduced SBmGW-PLBs into the pGEM-T vector and isolated 28 bacterial clones after transformation into *E. coli*. pGEM-T insert sequencing revealed that 13 of 28 indels (46%) restored the EGFP-ORF (Figure 1E). Four of these were 2-nt deletions (K2, K23, K33, and K34), of which three restored the wild-type sequence and one (K33) converted a Val codon into Ala. Although a monoclonal origin of the three wild-type indels cannot be excluded, clonal outgrowth was deemed unlikely in the absence of selection. The other ORFs included amino acid substitutions combined with acquisitions induced by 1-nt insertions or amino acid deletions combined with substitutions induced by 5′ nucleotide deletions (Figure 1E). Importantly, none of the recovered sequences contained the original SBmGW mutation, suggesting an on-target mutation rate approaching 100%.

To test whether non-canonical ORFs are compatible with EGFP fluorescence, we replicated recovered ORFs in SBGW vectors by site-specific mutagenesis and individually transfected these into HEK293T cells. After 48 hr, FACS analysis identified K16, K18, and K33 ORFs compatible with EGFP expression because most transfected BFP^+^ cells were also positive for EGFP. In contrast, single amino acid deletions from K6 and K29 ORFs were incompatible with EGFP expression (Figure 1F). This is consistent with previous reporting showing that N-terminal EGFP mutations abolish EGFP fluorescence.^25^

Overall, 25% of the indels repaired mEGFP—a frequency similar to the fraction of EGFP^+^ cells recovered from LC-sgEGFP2.3-transduced SBmGW-PLB cells (Figure 1B).

**Figure 2:**
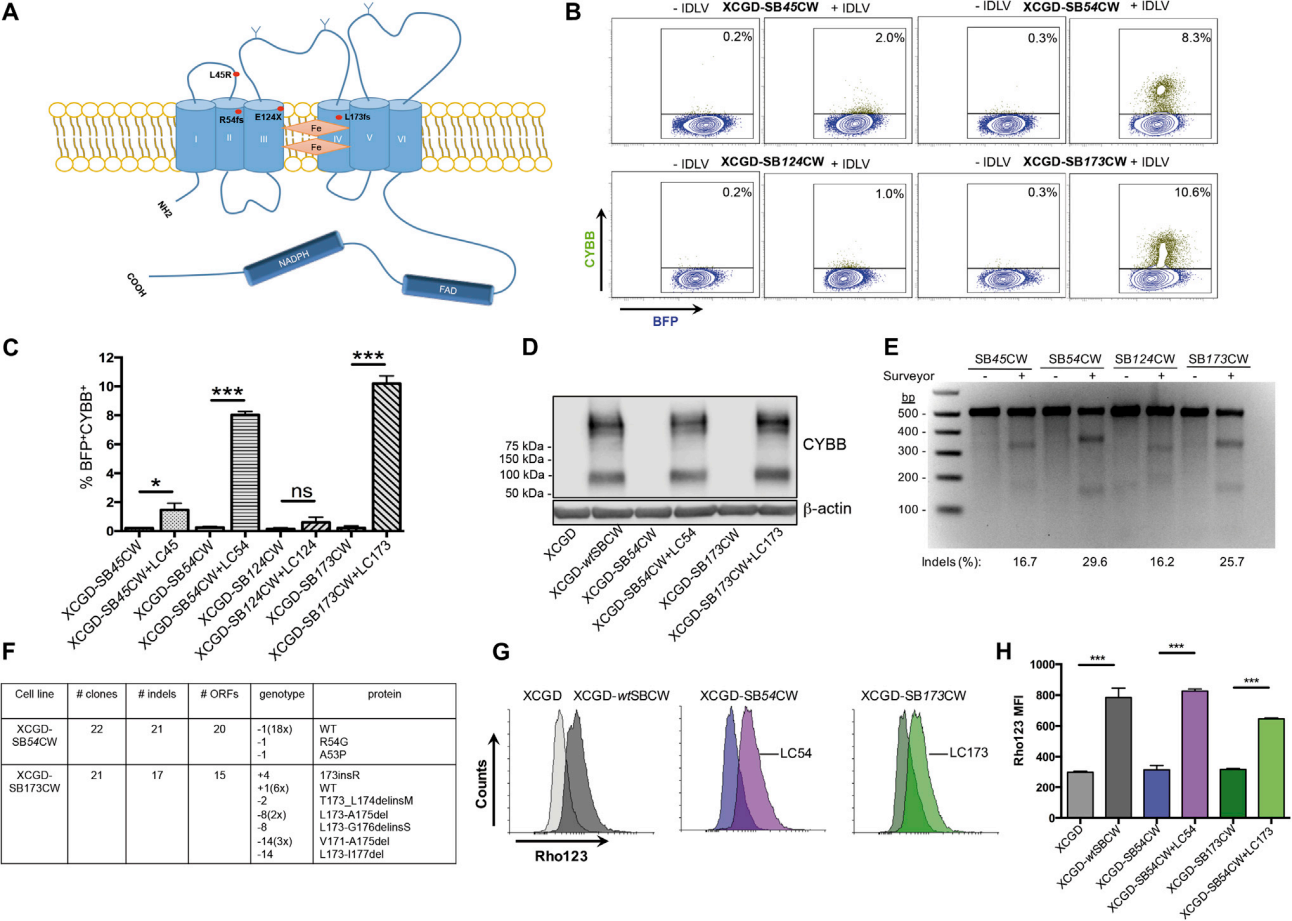
Repair of *CYBB* Mutations in XCGD-PLB Cells (A) Schematic representation of CYBB and positions of the selected disease mutations. (B) FACS profiles of sorted BFP^+^ XCGD cells stained with monoclonal 7D5 anti-human CYBB antibody 4 days after RGN transduction. (C) Frequency of CYBB^+^ cells among BFP^+^ XCGD cells. Results are represented as mean ± SD of 3 independent experiments. (D) Western blot showing CYBB expression in IDLV-transduced BFP^+^ XCGD cells harboring the different CYBB mutations. (E) Surveyor assay performed with *CYBB* PCR products of IDLV-treated XCGD cells. Indel frequency was calculated according to the formula published by Hsu et al.^39^ (F) Types of indels recovered from FACS-sorted CYBB^+^ XCGD cells. (G) Representative histograms depicting ROS production by differentiated, CYBB^+^ XCGD cells after stimulation with PMA. ROS levels were estimated by measuring the oxidative conversion of dihydrorhodamine 123 into rhodamine 123, which exhibits green fluorescence (DHR assay). (H) Mean fluorescence intensity induced by differentiated XCGD cells before and after IDLV transduction. XCGD and XCGD-SB*wt*CW cells served as negative and positive controls, respectively. Results are represented as mean ± SEM of 2 to 3 independent experiments. ***p < 0.001; *p < 0.05.

To test whether the donor template-free RGN-IDLV strategy would also correct *bone fide* disease mutations, we replaced EGFP in the SBGW vector with wild-type or mutated *CYBB* cDNAs. We generated 5 BFP-IRES-CYBB lentiviral vectors carrying either wild-type *CYBB* (SB*wt*CW) or one of the following XCGD patient-specific mutations: frameshift-*R54fs*CYBB (SB*54*CW), frameshift-*L173fs*CYBB (SB*173*CW), nonsense- *E124X*CYBB (SB*124*CW), or missense-*L45R*CYBB (SB*45*CW) (Figure 2A; Table S1).

Wild-type and mutant *CYBB-*carrying lentiviruses were transduced into *CYBB*-null PLB cells (XCGD cells)^17^ by low MOI infection and transduced XCGD cells were analyzed for BFP- and CYBB expression by flow cytometry 4 days post-infection. Most SB*wt*CW-transduced XCGD cells expressed both BFP and CYBB, whereas all XCGD cells carrying mutant copies expressed only BFP (Figure S3).

Next, we cloned sgRNAs targeting different *CYBB* mutations (Table S1) into pLentiCRISPRv2 and infected FACS-sorted, BFP^+^XCGD cells with corresponding LC-sgCYBB IDLVs. After 14 days, up to 10% of XCGD-*54*CW and XCGD-*173*CW cells stained positive for CYBB (Figures 2B and 2C) and expressed full-length CYBB, as revealed by western blotting (Figure 2D). Although repair efficiency was only half of that achieved for mEGFP in SBmGW-PLB cells (Figure 1B), so was the on-target mutation rate (Figure 2E). In contrast, less than 2% of XCGD-*124*CW and XCGD-SB*45*CW cells stained positive for CYBB (Figures 2B and 2C), suggesting that nonsense and missense mutations are less amenable to RGN repair. However, both mutations also showed reduced on-target mutation rates (Figure 2E), presumably caused by the low CG content of the respective sgRNA (Table S1).^26^ Moreover, Cas9 tolerance of single nucleotide mismatches^27^ could have selected against single nucleotide substitutions.

**Figure 3:**
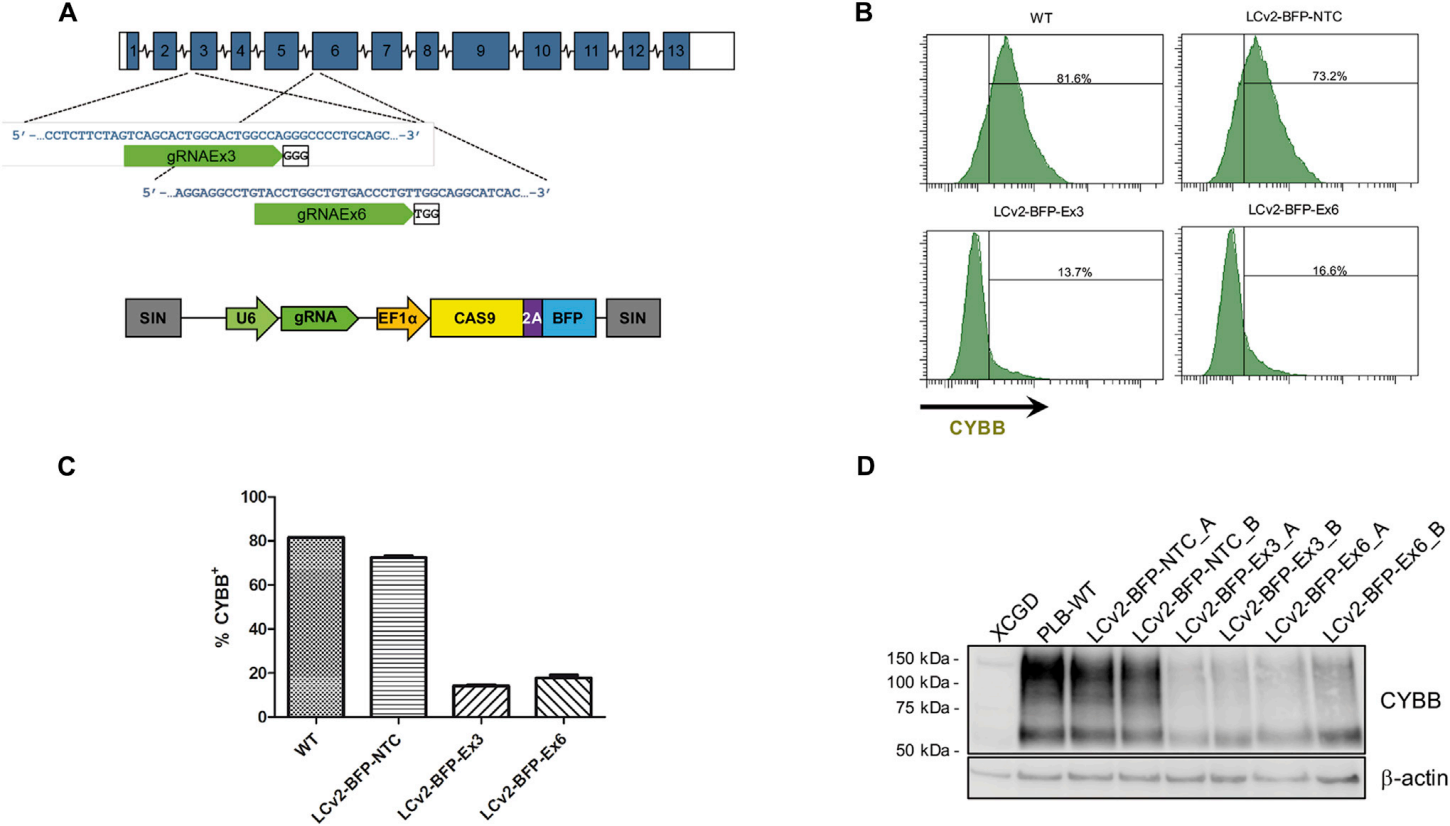
Targeting Efficiency at the Endogenous *CYBB* Locus (A) Lentiviral construct (bottom) used to target the endogenous CYBB locus (top) with the corresponding sgRNAs. (B) FACS profiles of CYBB-expressing PLB cells 14 days after RGN transduction. (C) Bar graph showing the frequency of CYBB^+^ cells after RGN transduction. Results are represented as mean ± SEM of 2 independent experiments. (D) Western blot showing reduced CYBB expression in RGN-transduced PLB cells. (A) and (B) denote independent experiments. Ex, exon; NTC, non-target control; WT, untreated control.

To indentify indels compatible with CYBB expression, we sequenced several indels from FACS-sorted CYBB^+^, XCGD-*54*CW, and XCGD-*173*CW cells recovered by shot-gun cloning. As anticipated, most indels from XCGD-*54*CW and XCGD-*173*CW mutations reconstituted the ORFs (Figures 2F and S4). For the XCGD-*54*CW mutation, only two ORFs with single nucleotide deletions failed to match the wild-type sequence. Although one of these might be compatible with CYBB expression because it retains Arg^54^ (K21, Figure S4), which is essential for reactive oxygen species (ROS) production, the other Arg54Gly mutation is likely non-functional.^28^ In contrast, over half of the ORFs reconstituting the XCGD*173*CW mutation residing in the 4^th^ transmembrane domain of CYBB (Figure 2A) were non-canonical, including one 4-nt insertion, one 2-nt deletion, three 8-nt deletions, and four 14-nt deletions (Figures 3F and S4). According to SMART, the *in silico* modular architecture research tool (http://smart.embl-heidelberg.de/), none of these ORFs appears to affect the integrity of the transmembrane domain, explaining why gene repair efficiency was highest in XCGD-*173*CW cells (Figures 2B and 2C).

Next, we PCR amplified 400–500 bp genomic DNA fragments from the top 4 off-target loci in IDLV-treated XCGD-54CW and XCGD-173CW cells (Figure S5A) and subjected these to the Surveyor assay. Consistent with a high sgRNA specificity, no cleavage was found in the off-targets of both RGN-transduced cell lines (Figure S5B).

To test whether CYBB expression is equivalent to gene repair, we estimated ROS production by differentiated CYBB^+^, XCGD-*54*CW, and XCGD-*173*CW cells using the dihydrorhodamine-123 (DHR) reduction assay.^29^ Figures 3B and 3G show *CYBB*-corrected XCGD-*54*CW and XCGD-*173*CW neutrophils obtained after DMSO induced differentiation (Figure S6), which produced superoxide equivalent to wild-type CYBB expressing (XCGD-SB*wt*CW) cells.

Finally, to determine whether the NHEJ gene repair strategy would have similar efficiency at the endogenous *CYBB* locus, we transduced wild-type PLB cells with LC-BFP-sgCYBB LVs, in which sgRNAs targeting R54fs and L173fs mutations were replaced with sgRNAs targeting corresponding wild-type sequences (sgEx3 and sgEx6; Figure 3A; Table S1). Non-transduced PLB cells and PLB cells transduced with LVs expressing scrambled (off-target) sgRNA (LC-NTC; Table S1) served as positive controls. Flow cytometry 4 days post-transduction, showed that about 60% of LC-sgEx3- and LC-sgEx6-LV transduced cells ceased to express CYBB (Figure 3B), which was confirmed by western blotting (Figure 3C). To directly estimate the on-target mutation rate, we sequenced several indels recovered by shot-gun cloning from LC-sgEx3- and LC-sgEx6-LV transduced cells. As shown in Figure S7, 21 out of 25 exons 3 and 19 out of 25 exons 6 exhibited a mutation, suggesting an on-target mutation rate of over 75%. Assuming gene repair efficiencies of about 25%, we predict an *in situ* gene repair efficiency of at least 18%, which is sufficient to protect X-CGD patients from microbial infections.^30^, ^31^

Although these observations require validation with patient material, they nevertheless demonstrate that frameshift mutations can be effectively repaired in hematopoietic cells by donor-template free CRISPR/Cas9 technology. According to the CYBBbase database (http://structure.bmc.lu.se/idbase/CYBBbase/browser.php?content=browser), 24% of XCGD patients harbor *CYBB* frameshift mutations. Because this frequency is similar to the frequency of frameshift mutations recovered in the *IL2Rγ*^32^ (http://www.ncbi.nlm.nih.gov/lovd/home.php?select_db=IL2RG), *WASP*^33^ (http://pidj.rcai.riken.jp/waspbase/), *ADA* (http://structure.bmc.lu.se/idbase/ADAbase/index.php?content=pubs/Idbases) and *HBB*^34^ (http://globin.cse.psu.edu/globin/hbvar/) genes of patients with X-linked immunodeficiency disease (X-SCID), Wiskott-Aldrich Syndrome, adenosinedeaminase immunodeficiency disease (ADA-SCID), and β-thalassemia, respectively, one in four of these patients is likely to benefit from gene therapy with donor template-free, RNA-guided Cas9 endonucleases.

## Materials and Methods

### Vectors and Endonucleases

Cas9 and gRNA_Cloning vector used for the nucleofection experiments were purchased from Addgene (plasmids #41815 and #41824).^35^ The gRNA_Cloning vector was optimized for gRNA cloning and expression by inserting the partially missing U6 promoter and gRNA scaffold sequences into the *SpeI* and *NdeI* sites of the gRNA_Cloning vector.^35^ The EGFP-targeting sgRNAs obtained by annealing the BH001–BH004 and BH037–BH044 oligonucleotides (Table S1) were cloned into the modified gRNA_Cloning vector using the Golden Gate Protocol.^36^

The dicistronic BFP/EGFP and BFP/CYBB lentiviral vectors were generated by first inserting EGFP- and CYBB cDNAs into *MscI* and *SbfI* sites of the TagBFP-expressing lentiviral vector -pHR’SINcPPT-SBW obtained from M. Grez.^37^ Subsequently, an ECMV-IRES was cloned into the *MscI* site between the BFP/EGFP or BFP/CYBB cDNAs.

EGFP mutations were introduced by inserting annealed synthetic oligonucleotides into the BstXI sites of EGFP (Oligos SFHR037, SFHR038, and SFHR046–SFHR055). CYBB mutations were introduced by standard site-specific mutagenesis using the BH137–BH144 primers (Table S1).

pLentiCRISPRv2 vectors containing the different sgRNAs were obtained by target-specific oligonucleotide annealing (Table S1) using the GoldenGate protocol.

### Cell Culture

Hematopoietic cells were grown in RPMI-1640 medium supplemented with 10% (v/v) heat-inactivated fetal calf serum (FCS), 2 mM glutamine, 100 U/mL penicillin, and 100 μg/mL streptomycin.

### Lentiviral Vector Production

Cell-free, lentiviral supernatants were produced by polyethylenimine (PEI) based transient co-transfection of HEK293T cells. Briefly, pLentiCRISPRv2 vectors, lentiviral *gag*/*pol* helper plasmids for integrating (pCMV8.91) or integrase defective (pCMV8.74) variants and envelope plasmid encoding the glycoprotein of vesicular stomatitis virus (VSV-G) (pMD2.G, Addgene #12259) were transfected at a molar ratio of 3:1:1 by standard PEI transfection. 48 hr post transfection, viral supernatants were harvested, sterile filtered (0.45-μm pore-size PVDF-membrane filter; Millipore, Schwalbach, Germany), and concentrated (60- to 100-fold) by ultracentrifugation over a 20% (w/v) sucrose cushion (50,000 × *g*, 2 hr, 4°C). Pelleted vector particles were resuspended in StemSpan SFEM serum-free medium (STEMCELL Technologies, Grenoble, France) without any supplements and stored at −80°C. SBGW lentiviral titers were determined in serial dilutions of viral supernatant by transduction of PLB-985 cells, followed by flow cytometry 4 to 5 days post-transduction. For determining IDLV titers, p24 viral coat protein concentrations were estimated in the viral supernatants using the p24 ELISA kit from INNOTEST (Fujirebio, Hannover, Germany) according to the manufacturer’s instructions. To determine the number of particles corresponding to every picogram of p24 antigen, we used a conversion factor of 6.12 × 10^3^ particles/pg derived from the flow cytometric analysis of PLB cells 48 hr after SBmGW-IDLV transduction.

### Flow Cytometry and Cell Sorting

For flow cytometry, cells were washed and resuspended in PBS. For cell surface antigen staining, cells were incubated in the dark with fluorescein isothiocyanate (FITC)-conjugated anti-flavocytochrome b558 7D5 (D162-4) (MBL International, Woburn, MA, USA) or CD11b-APC (Miltenyi Biotec no. 130-098-088) antibodies for 20–30 minutes at room temperature. Data acquisition was performed with a BD LSRFortessa flow cytometer (BD Biosciences, Heidelberg, Germany). Data were analyzed with BD FACSDiva software (BD Biosciences, Heidelberg, Germany) or flowing software 2.5.1. Cell sorting was performed in a BD FACSAria III flow cytometer (BD Biosciences, Heidelberg, Germany).

### Molecular Analyses

For indel identification, PCR products were generated using primers SFHR020/SFHR043 for exogenous EGFP, BH229/BH234 for exogenous CYBB, SFHR128/SFHR129 for endogenous Ex3, and SFHR132/SFHR133 for endogenous Ex6 (Table S1). Shot gun cloning of PCR amplification products was performed using the pGEM-T vector system (Promega, Mannheim, Germany) according to the manufacturer’s instructions. pGEM-T inserts derived from individual bacterial colonies were sequenced using the standard M13-forward primer.

### Western Blot

Cells were lysed for 30 min on ice in lysis buffer (50 mM Tris, pH 7.4, 0.15 M NaCl, 2 mM EDTA, and 1% NP-40) supplemented with Protease Inhibitor Cocktail (Roche, Mannheim, Germany). The samples were resuspended in loading buffer containing 20% beta-mercaptoethanol, boiled 5 min at 95°C, and separated by SDS-PAGE. Mouse monoclonal antibody against GFP was purchased from Roche, and rabbit monoclonal antibody against GAPDH was purchased from Cell Signaling (Frankfurt am Main, Germany). Monoclonal anti-human CYBB antibody (moAB48) was obtained from LifeSpan BioSciences (Seattle, WA, USA).

### Surveyor Assay

RGN-targeted CYBB sites were PCR amplified to obtain 500-nt-sized products using the BH229/BH230, BH231/BH232, and BH233/BH234 primers (Table S1). For the analysis of on-target mutation rates, 400 ng PCR product was subjected to the Surveyor assay using the Surveyor Mutation Detection Kit-S100 (IDT, Leuven, Belgium) and the manufacturer’s instructions. Indel percentages were derived from ImageJ plots^38^ using the following formula: 100 × (1 − (1 − (b + c)/(a + b + c))^0.5^), where a is the integrated intensity of the undigested PCR product, and b and c are the integrated intensities of each cleavage product.

### DHR Reduction Assay

DHR assays were performed as described by Brendel et al.^29^ Briefly, for granulocytic differentiation, cells were plated at a concentration of 2 × 10^5^ cells/mL in RPMI-1640 supplemented with 2.5% heat inactivated FCS, 2 mM glutamine, 100 U/mL penicillin, and 100 μg/mL streptomycin and 1.25% dimethyl sulfoxide (Sigma-Aldrich, Taufkirchen, Germany) for at least 7 days. For estimating ROS production, the differentiated cells were suspended in 1 mL pre-warmed Hank’s balanced salt solution (HBSS) (Life Technologies, Darmstadt, Germany), supplemented with 7.5 mmol/L D-Glucose, 0.5% bovine serum albumin (BSA) (Sigma-Aldrich), 2,000 U/mL catalase (Sigma-Aldrich), and 5 μg/mL DHR123 (Sigma-Aldrich). Following incubation for 10 min at 37°C, cells were exposed to 0.1 μmol/L phorbol 12-myristate 13-acetate (PMA; Sigma-Aldrich) for 15 min and placed on ice. Rhodamin 123 fluorescence was measured in a flow cytometer within the next 30 min.

### Statistical Analysis

For statistical comparisons between groups, Student’s t test or one-way ANOVA with Bonferoni post hoc test were used as appropriate in conjunction with GraphPad Prism 5 software.

## Author Contributions

D.S., J.S., A.T., R.E., S.S., N.K., and F.S. conducted the experiments. D.S., H.v.M., and F.S. conceived the study, designed the experiments, and wrote the paper.

## Supplemental Information

Document S1. Figures S1–S6 and Tables S1 and S2

Document S2. Article plus Supplemental Information
