# Supplementary material for: High Efficiency Gene Correction in Hematopoietic Cells by Donor-Template-Free CRISPR/Cas9 Genome Editing
Source: Mol Ther Nucleic Acids. 2017 Nov 10;10:1–8. doi: 10.1016/j.omtn.2017.11.001 (PMC5723376; doi:10.1016/j.omtn.2017.11.001)
Supplement: Document S1. Figures S1–S6 and Tables S1 and S2 [file mmc1.pdf]

OMTN, Volume *10*

Supplemental Information

High Efficiency Gene Correction in Hematopoietic Cells by Donor-Template-Free CRISPR/Cas9 Genome Editing

Duran Sürün, Joachim Schwäble, Ana Tomasovic, Roy Ehling, Stefan Stein, Nina Kurrle, Harald von Melchner, and Frank Schnütgen

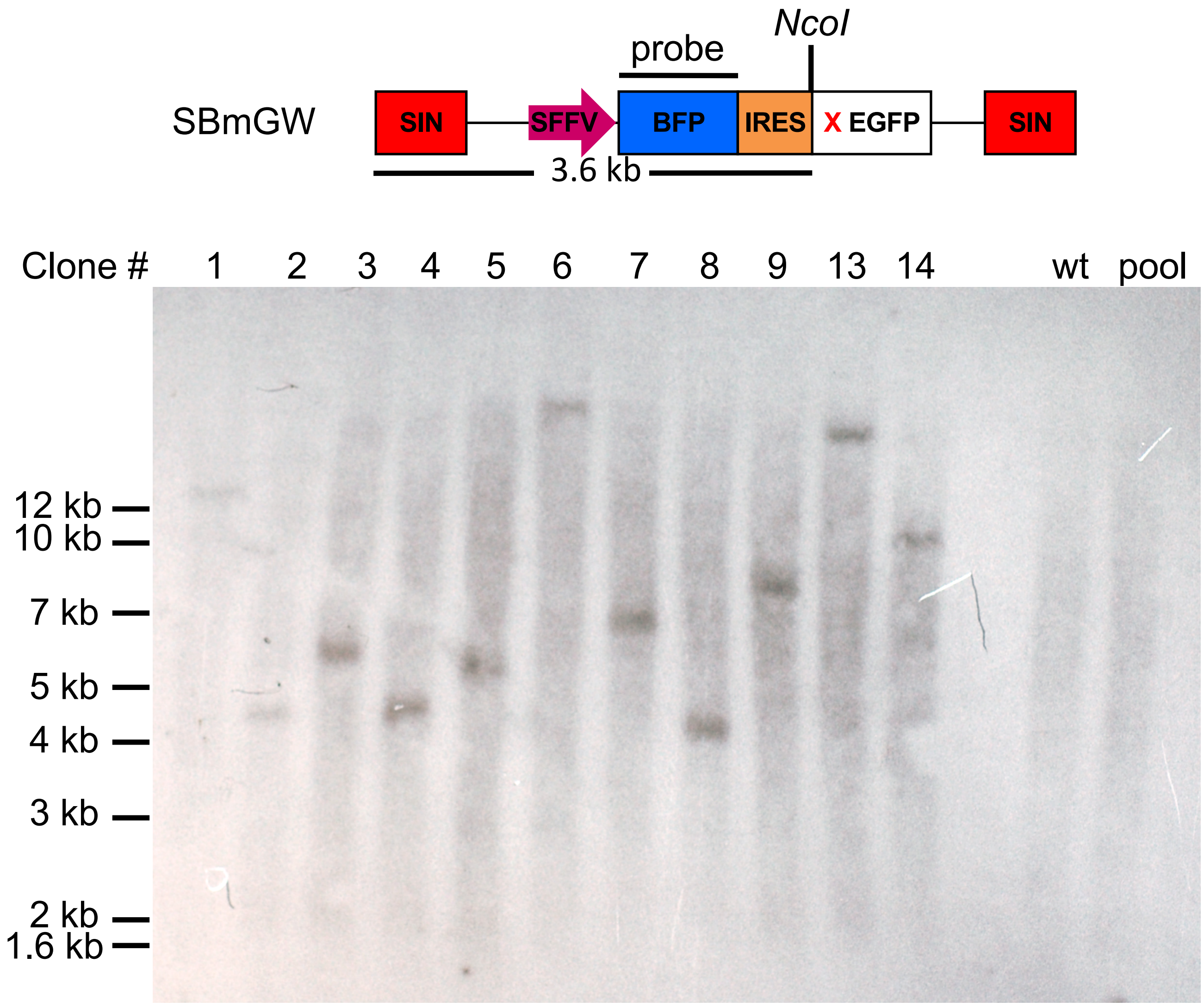

Supplemental Figure 1: Single copy integration of SBmGW lentiviruses in PLB cells. BFP+ SBmGW-PLB clones were isolated by limiting dilution and subjected to Southern blot analysis. *NcoI* restriction fragments of genomic DNAs of individual clones were resolved by agarose gel electrophoresis, transferred onto nylon mebranes and hybridized to a ^{32}P-labeld BFP probe.

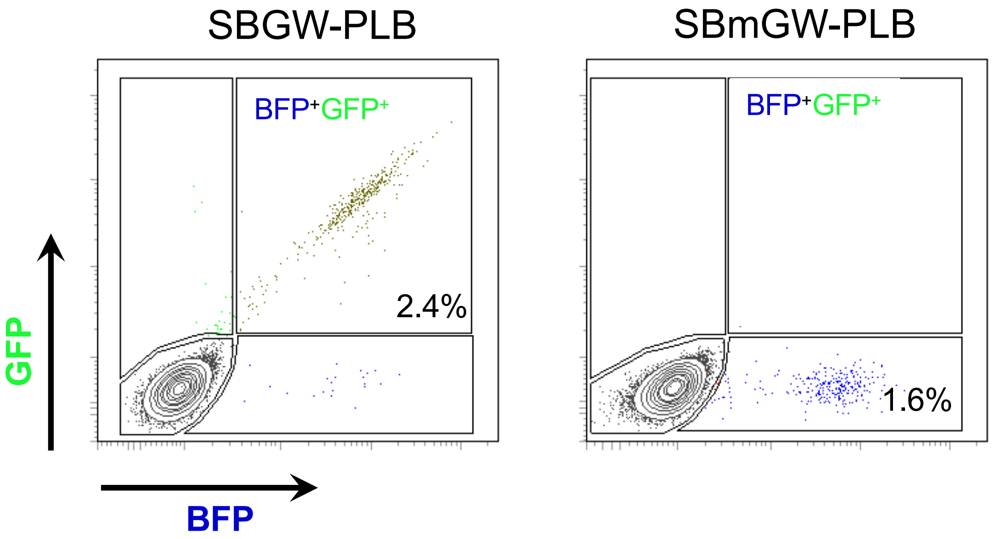

Supplemental Figure 2: FACS profiles of SBGW- and SBmGW-PLB reporter cells

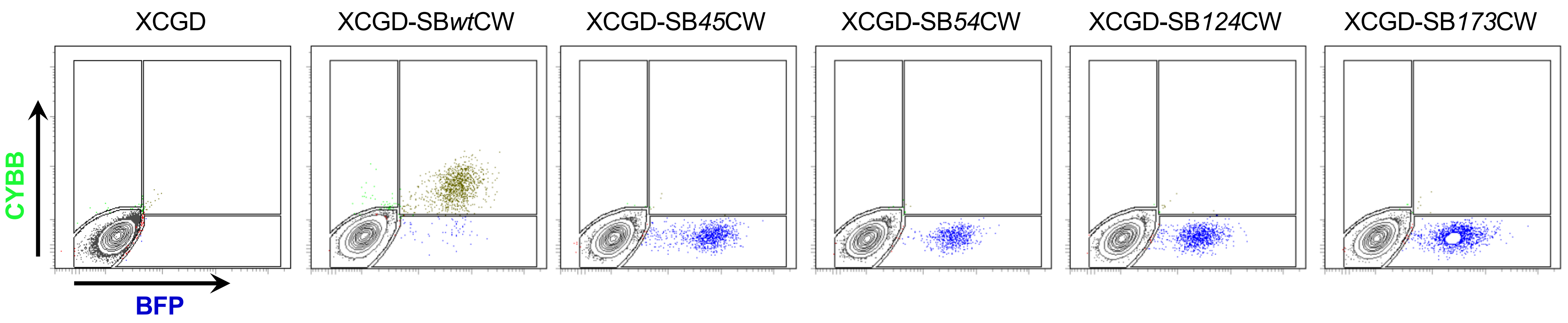

Supplemental Figure 3: FACS profiles of XCGD cells expressing wild-type and mutant CYBB cDNAs. Note that only XCGD-SB*wt*CW cells expressing wild type *CYBB* are double positive for BFP and CYBB.

		Genotype
SB*54*CW	CCCGGAAGCTGCTGGGCTCAGCACTGGCACTGGCCCAGGGCCCCTGCCGCCTGCCTGAAC	
K1	CCCGGAAGCTGCTGGGCTCAGCACTGGCACTGG-CCAGGGCCCCTGCCGCCTGCCTGAAC	-1
K2	CCCGGAAGCTGCTGGGCTCAGCACTGGCACTGG-CCAGGGCCCCTGCCGCCTGCCTGAAC	-1
K3	CCCGGAAGCTGCTGGGCTCAGCACTGGCACTGG-CCAGGGCCCCTGCCGCCTGCCTGAAC	-1
K4	CCCGGAAGCTGCTGGGCTCAGCACTGGCACTGG-CCAGGGCCCCTGCCGCCTGCCTGAAC	-1
K5	CCCGGAAGCTGCTGGGCTCAGCACTGGCACTGGCCCAGGGCCCCTGCCGCCTGCCTGAAC	0
K6	CCCGGAAGCTGCTGGGCTCAGCACTGGCACTGG-CC**G**GGGCCCCTGCCGCCTGCCTGAAC	-1 (A>G)
K7	CCCGGAAGCTGCTGGGCTCAGCACTGGCACTGG-CCAGGGCCCCTGCCGCCTGCCTGAAC	-1
K8	CCCGGAAGCTGCTGGGCTCAGCACTGGCACTGG-CCAGGGCCCCTGCCGCCTGCCTGAAC	-1
K9	CCCGGAAGCTGCTGGGCTCAGCACTGGCACTGG-CCAGGGCCCCTGCCGCCTGCCTGAAC	-1
K10	CCCGGAAGCTGCTGGGCTCAGCACTGGCACTGG-CCAGGGCCCCTGCCGCCTGCCTGAAC	-1
K11	CCCGGAAGCTGCTGGGCTCAGCACTGGCACTGG-CCAGGGCCCCTGCCGCCTGCCTGAAC	-1
K12	CCCGGAAGCTGCTGGGCTCAGCACTGGCACTGG-CCAGGGCCCCTGCCGCCTGCCTGAAC	-1
K13	CCCGGAAGCTGCTGGGCTCAGCACTGGCACTGG-CCAGGGCCCCTGCCGCCTGCCTGAAC	-1
K14	CCCGGAAGCTGCTGGGCTCAGCACTGGCACTGG-CCAGGGCCCCTGCCGCCTGCCTGAAC	-1
K15	CCCGGAAGCTGCTGGGCTCAGCACTGGCACTGG-CCAGGGCCCCTGCCGCCTGCCTGAAC	-1
K16	CCCGGAAGCTGCTGGGCTC---------------CCAGGGCCCCTGCCGCCTGCCTGAAC	-15
K17	CCCGGAAGCTGCTGGGCTCAGCACTGGCACTGG-CCAGGGCCCCTGCCGCCTGCCTGAAC	-1
K18	CCCGGAAGCTGCTGGGCTCAGCACTGGCACTGG-CCAGGGCCCCTGCCGCCTGCCTGAAC	-1
K19	CCCGGAAGCTGCTGGGCTCAGCACTGGCACTGG-CCAGGGCCCCTGCCGCCTGCCTGAAC	-1
K20	CCCGGAAGCTGCTGGGCTCAGCACTGGCACTGG-CCAGGGCCCCTGCCGCCTGCCTGAAC	-1
K21	CCCGGAAGCTGCTGGGCTCAGCACTGGCACTG**-**CCCAGGGCCCCTGCCGCCTGCCTGAAC	-1
K22	CCCGGAAGCTGCTGGGCTCAGCACTGGCACTGG-CCAGGGCCCCTGCCGCCTGCCTGAAC	-1

		Genotype
SB*173*CW	CCCCGAGGGCGGCCTGTACCTGGCTGTGACC----TGTTGGCCGGCATCACCGGCGTGGT	
K1	CCCCGAGGGCGGCCTGTACCTGGCTGTGACC----TGTTGGCCGGCATCACCGGCGTGGT	0
K2	CCCCGAGGGCGGCCTGTACCTG------------------GCCGGCATCACCGGCGTGGT	-14
K3	CCCCGAGGGCGGCCTGTACCTG------------------GCCGGCATCACCGGCGTGGT	-14
K4	CCCCGAAGGCGGCCTGTACCTGGCTGTGA------------CCAGCATCACCGGCGTGGT	-8
K5	CCCCGAGGGCGGCCTGTACCTGGCTGTGA------TGTTGGCCGGCATCACCGGCGTGGT	-6
K6	CCCCGAGGGCGGCCTGTACCTGGCTG------------TGGCCGGCATCACCGGCGTGGT	-8
K7	CCCCGAGGGCGGCCTGTACCTGGCTGTGACCC---TGTTGGCCGGCATCACCGGCGTGGT	+1
K8	CCCCGAGGGCGGCCTGTACCTGGCTGTGACCC---TGTTGGCCGGCATCACCGGCGTGGT	+1
K9	CCCCGAGGGCGGCCTGTACCTGGCTGTGA------------CCGGCATCACCGGCGTGGT	-8
K10	CCCCGAGGGCGGCCTATACCTG-------------TGTTGGCCGGCATCACCGGCGTGGT	-9
K11	CCCCGAGGGCGGCCTGTACCTGGCTGTGACCAGGCTGTTGGCCGGCATCACCGGCGTGGT	+4
K13	CCCCGAGGGCGGCCTGTACCTGGCTGTGACC----TGTTGGCCGGCATCACCGGCGTGGT	0
K14	CCCCGAGGGCGGCCTGTACCTGGCTGTGACCC---TGTTGGCCGGCATCACCGGCGTGGT	+1
K15	CCCCGAGGGCGGCCTGTACCTGGCTGTGACC----TGTTGGCCGGCATCACCGGCGTGGT	0
K16	CCCCGAGGGCGGCCTGTACCTGGCTGTGA------TGTTGGCCGGCATCACCGGCGTGGT	-2
K17	CCCCGAGGGCGGCCTGTACCTGGCTGTGACCC---TGTTGGCCGGCATCACCGGCGTGGT	+1
K18	CCCCGAGGGCGGCCTGTACCTGGCTGTGAC------------------CACCGGCGTGGT	-14
K19	CCCCGAGGGCGGCCTGTACCTGGCTGTGACCT---TGTTGGCCGGCATCACCGGCGTGGT	+1
K20	CCCCGAGGGCGGCCTGTACCTG------------------GCCGGCATCACCGGCGTGGT	-14
K21	CCCCGAGGGCGGCCTGTACCTGGCTGTGACC----TGTTGGCCGGCATCACCGGCGTGGT	0
K22	CCCCGAGGGCGGCCTGTACCTGGCTGTGACCC---TGTTGGCCGGCATCACCGGCGTGGT	+1

Supplementary Figure 4 : Indel sequences recovered by shot gun cloning from XCGD-SB*54*CW and XCGD-SB*173*CW cells.

A

GTACCTGGCTGTGACCTGT**TGG**	sgCYBB54
CAGCACAGACACTGGCCCA**AAG**	chr14:+32749541
CATCACTGGCTCTGGCCCA**TAG**	chr12:-103703290
CAACAGGGGCACTGGCCCA**TGG**	chr1:+204799512
TGGCAGTGGCACTGGCCCA**GGG**	chr19:-46999128
CAGCACTGGCACTGGCCCA**GGG**	sgCYBB173
TTACCTTGCTGTGACCTGT**TAG**	chr2:+186140924
GTTCCTGGCTGTGATCTGT**GAG**	chr7:+73675051
AAACCTAGCTGTGACCTGT**AAG**	chrX:-128220322
CCAACTTGCTGTGACCTGT**GGG**	chr18:+24746359

B

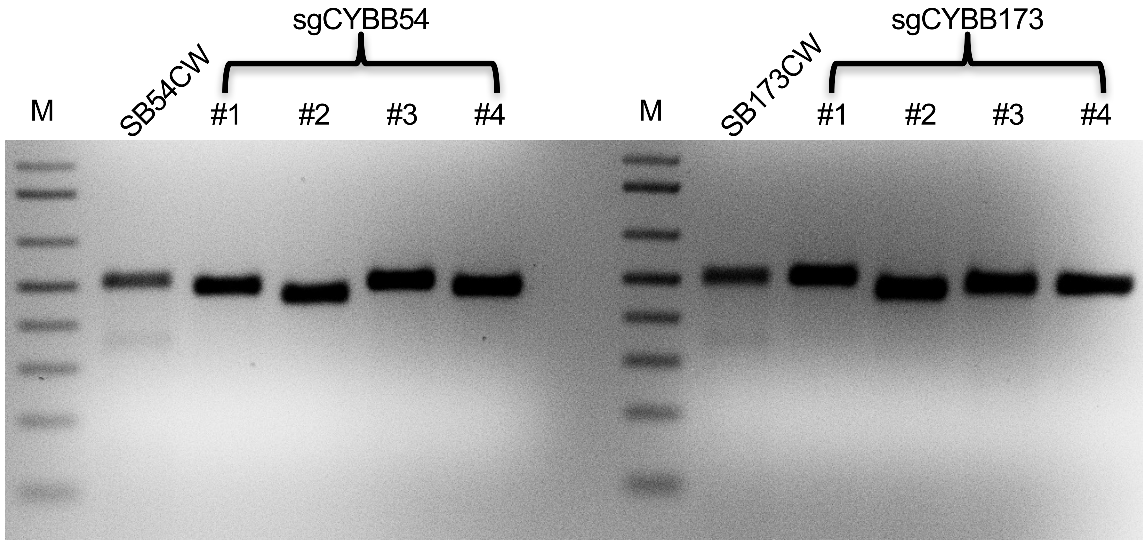

Supplemental Figure 5: Off target analysis in IDLV treated XCGD-54CW and XCGD-173CW cells. **A.** Top 4 off targets for sgCYBB54 and sgCYBB173 RNAs selected from the benchling.com website. **B.** Surveyor assay

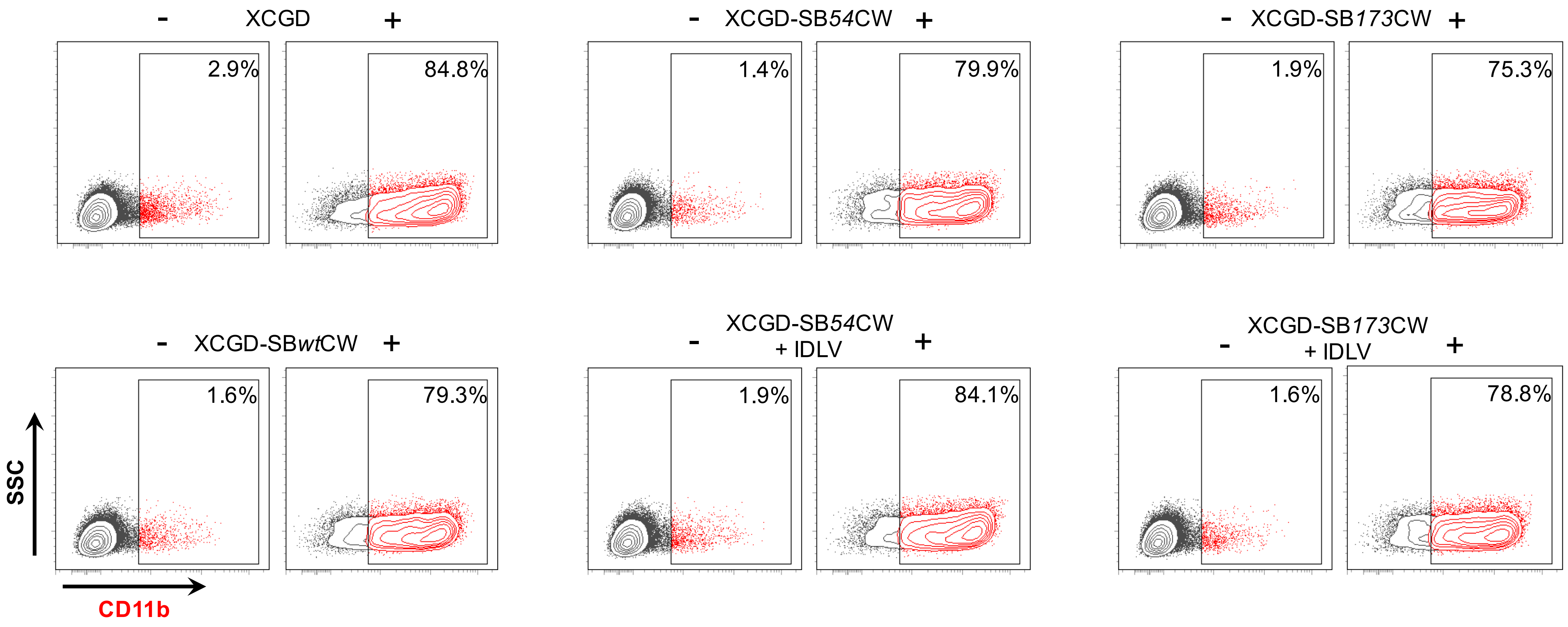

Supplemental Figure 6: Differentiation of XCGD cells after exposure to DMSO. Cells were incubated for 7 days with DMSO (+), stained with anti-CD11b antibody and analyzed by FACS.

		Genotype
Ex3	C GCC TCT TC TAG TCA GC ACT GGC AC TGG CCA **GGG**CC CCT GC AGC CTG CC TGA ATT TC AAC	
C1	C GCC TCT TC TAG TCA GC ACT GGC AC --- --- -- --- --T GC AGC CTG CC TGA ATT TC AAC	-13
C2	C GCC TCT TC TAG TCA GC ACT GGC AC TG- CCA GG GCC CCT GC AGC CTG CC TGA ATT TC AAC	-1
C3	C GCC TCT TC TAG TCA GC ACT GGC AC TG- CCA GG GCC CCT GC AGC CTG CC TGA ATT TC AAC	-1
C4	C GCC TCT TC TAG TCA GC ACT GGC AC TGG CCA GG GCC CCT GC AGC CTG CC TGA ATT TC AAC	0
C5	C GCC TCT TC TAG TCA GC ACT GGC AC TG- CCA GG GCC CCT GC AGC CTG CC TGA ATT TC AAC	-1
C6	C GCC TCT TC TAG TCA GC ACT GGC AC TGG CT- -- --- --- -- --- --- -- --- --- -- ---	-166
C7	C GCC TCT TC TAG TCA GC ACT GG- -- --- CCA GG GCC CCT GC AGC CTG CC TGA ATT TC AAC	-6
C8	C GCC TCT TC TAG TCA GC ACT G-- -- --- --- -- -CC CCT GC AGC CTG CC TGA ATT TC AAC	-13
C9	C GCC TCT TC TAG TCA GC ACT GGC A- --- --- -- --- --- -- --- CTG CC TGA ATT TC AAC	-20
C10	C GCC TCT TC TAG TCA GC ACT GGC AC TGG CCA GG GCC CCT GC AGC CTG CC TGA ATT TC AAC	0
C11	C GCC TCT TC TAG TCA GC ACT GGC AC TG- CCA GG GCC CCT GC AGC CTG CC TGA ATT TC AAC	-1
C12	C GCC TCT TC TAG TCA GC ACT GGC AC TG- -CA GG GCC CCT GC AGC CTG CC TGA ATT TC AAC	-2
C13	C GCC TCT TC TAG TCA GC ACT GGC AC TGG --- -- --- --- -- --- -TG CC TGA ATT TC AAC	-17
C14	C GCC TCT TC TAG TCA GC ACT GGC AC TA- CCA GG GCC CCT GC AGC CTG CC TGA ATT TC AAC	-1
C15	C GCC TCT TC TAG TCA GC ACT GGC AC TG- CCA GG GCC CCT GC AGC CTG CC TGA ATT TC AAC	-1
C16	- --- --- -- --- --- -- --- --- -- --- --- -- --- --- -- --- --- -- --- --- -- -AC	-120
C17	C GCC TCT TC TAG TCA GC ACT GGC AC TG- CCA GG GCC CCT GC AGC CTG CC TGA ATT TC AAC	-1
C18	C GCC TCT TC TAG TCA GC ACT GGC AC TG- CCA GG GCC CCT GC AGC CTG CC TGA ATT TC AAC	-1
C19	C GCC TCT TC TAG TCA GC ACT GGC AC --- --- -- --- --T GC AGC CTG CC TGA ATT TC AAC	-14
C20	C GCC TCT TC TAG TCA GC ACT GGC AC TG- CCA GG GCC CCT GC AGC CTG CC TGA ATT TC AAC	-1
C21	C GCC TCT TC TAG TCA GC ACT GGC AC TGG CCA GG GCC CCT GC AGC CTG CC TGA ATT TC AAC	0
C22	C GCC TCT TC TAG TCA GC ACT GGC AC T-- CCA GG GCC CCT GC AGC CTG CC TGA ATT TC AAC	-2
C23	C GCC TCT TC TAG TCA GC ACT GGC AC TGG CCA GG GCC CCT GC AGC CTG CC TGA ATT TC AAC	0
C24	C GCC TCT TC TAG TCA GC ACT GGC AC TG- --- -- GCC CCT GC AGC CTG CC TGA ATT TC AAC	-6
C25	C GCC TCT TC TAG TCA GC ACT GGC AC TG- CCA GG GCC CCT GC AGC CTG CC TGA ATT TC AAC	-1

		Genotype
Ex6	C TGA AGG AG GCC TGT AC CTG GCT GT GAC CCT GT **TGG** CAG GC ATC ACT GG AGT TGT CA TCA	
C1	C TGA AGG AG --- --- -- --- --- -- --- --- -- --G CAG GC ATC ACT GG AGT TGT CA TCA	-26
C2	C TGA AGG AG GCC TGT AC CTG GCT GT GAC CCT GT TGG CAG GC ATC ACT GG AGT TGT CA TCA	0
C3	C TGA AGG AG GCC TGT AC CTG GCT GT --- --- -- --- --- GC ATC ACT GG AGT TGT CA TCA	-14
C4	C TGA AGG AG GCC TGT AC CTG GCT GT GAC CCT GT TGG CAG GC ATC ACT GG AGT TGT CA TCA	0
C5	C TGA AGG AG GCC TGT AC CTG G-- -- --- --- -- --- CAG GC ATC ACT GG AGT TGT CA TCA	-15
C6	C TGA AGG AG GCC TGT AC CTG GCT GT GAC CC- GT TGG CAG GC ATC ACT GG AGT TGT CA TCA	-1
C7	C TGA AGG AG GCC TGT AC CTG --- -- --- --- -- --- --- -- --- --- -- --- --- -- ---	-47
C8	C TGA AGG AG GCC TGT AC CAG --- -- --- --- -- --- --- GC ATC ACT GG AGT TGT CA TCA	-19
C9	C TGA AGG -- --- --- -- --- --- -- --- --- -- -TA CAG GC ATC ACT GG AGT TGT CA TCA	-27
C10	C TGA AGG AG GCC TGT AC CTG GCT GT GAC CCT GT TGG CAG GC ATC ACT GG AGT TGT CA TCA	0
C11	C TGA AGG AG GCC TGT AC CTG GCT GT GAC CCT GT TGG CAG GC ATC ACT GG AGT TGT CA TCA	0
C12	C TGA AGG AG GCC TGT AC CTG GCT GT GAC CC- GT TGG CAG GC ATC ACT GG AGT TGT CA TCA	-1
C13	C TGA AGG AG GCC TGT AC CTG T-- -- --- --- GT TGG CAG GC ATC ACT GG AGT TGT CA TCA	-10
C14	- --- --- -- --- --- -- --- --- -- --- --- -- --- --- -- --- --- -- --- --- -- ---	-198
C15	C TGA AGG AG GCC TGT AC CTG G-- -- --- --- -- --- CAG GC ATC ACT GG AGT TGT CA TCA	-16
C16	C TGA AGG AG GCC TGT AC CTG GCT GT GAC CC- -- ATG CAG GC ATC ACT GG AGT TGT CA TCA	-3
C17	C TGA AGG AG GCC TGT AC CTG GCT GT G-- --- -- --- --- -A ATC ACT GG AGT TGT CA TCA	-14
C18	C TGA AGG AG GCC TGT AC CTG GCT GT GAC CC- GT TGG CAG GC ATC ACT GG AGT TGT CA TCA	-1
C19	C TGA AGG AG GCC TGT AC CTG GCT GT GAC CCT GT TGG CAG GC ATC ACT GG AGT TGT CA TCA	0
C20	C TGA AGG AG GCC TGT AC CTG GC- -- --- --T GT TGG CAG GC ATC ACT GG AGT TGT CA TCA	-8
C21	C TGA AGG AG GCC TGT AC CTG GCT GT --- --T GT TGG CAG GC ATC ACT GG AGT TGT CA TCA	-5
C22	C TGA AGG AG GCC TGT AC CTG GCT GT GAC CC- GT TGG CAG GC ATC ACT GG AGT TGT CA TCA	-1
C23	C TGA AGG AG GCC TGT AC CTG GCT GT GAC CC- -- --- --G GC ATC ACT GG AGT TGT CA TCA	-8
C24	C TGA AGG AG GCC TGT AC CTG GCT GT GAC CCT GT TGG CAG GC ATC ACT GG AGT TGT CA TCA	0
C25	C TGA AGG AG GCC TGT AC CTG GCT G- --- --T GT TGG CAG GC ATC ACT GG AGT TGT CA TCA	-6

Supplemental Figure 7: Indel sequences recovered by shot gun cloning from LC-sgEx3- and LC-sgEx6-LV transduced cells.

Supplemental Table 1: Oligo nucleotides used in this study

**Oligo**		**Sequence (5'-3')**
**Cloning of EGFP sgRNAs**		
BH001	g32DG1 s	CACCGGAGGAACTGTTCACCGGCG
BH002	g32DG1 as	AAACGTGAACAGTTCCTCGCCCTC
BH003	g32DG2 s	CACCGGAGGAACTGTTCACCGGCG
BH004	g32DG2 as	AAACCGCCGGTGAACAGTTCCTCC
BH037	g32DG1.4 s	CACCGGGGCGAGGAACTGTTTCAC
BH038	g32DG1.4 as	AAACGTGAAACAGTTCCTCGCCCC
BH039	g32DG1.7 s	CACCGAAGGGCGAGGAACTGTCAC
BH040	g32DG1.7 as	AAACGTGACAGTTCCTCGCCCTTC
BH041	g32DG2.3 s/ sgEGFP2.3 s	CACCGGGAACTGTTCACCGCGGCG
BH042	g32DG2.3 as/ sgEGFP2.3 as	AAACCGCCGCGGTGAACAGTTCCC
BH043	g32DG2.5 s	CACCGCGAGGAACTGTTCACGGCG
BH044	g32DG2.5 as	AAACCGCCGTGAACAGTTCCTCGC
**Cloning of CYBB sgRNAs**		
BH137	sgCYBB54 s	CACCGCAGCACTGGCACTGGCCCA
BH138	sgCYBB54 as	AAACTGGGCCAGTGCCAGTGCTGC
BH139	sgCYBB173 s	CACCGGTACCTGGCTGTGACCTGT
BH140	sgCYBB173 as	AAACACAGGTCACAGCCAGGTACC
BH141	sgCYBB45 s	CACCGTTTACACAAGAAAACGTCT
BH142	sgCYBB45 as	AAACAGACGTTTTCTTGTGTAAAC
BH143	sgCYBB124 s	CACCGACATCTATTTAATGTGTAA
BH144	sgCYBB124 as	AAACTTACACATTAAATAGATGTC
**Generation of EGFP mutant vectors**		
SFHR037	SBmGW s	ATGGTGTCCAAGGGCGAGGAACTGTTCACCGCGGCGTGGTGCCCATCCTG
SFHR038	SBmGW as	ATGGGCACCACGCCGCGGTGAACAGTTCCTCGCCCTTGGACACCATGGTT
SFHR046	SBmGW-K6 s	ATGGTGTCCAAGGGCGAGGAACTGTTCACCGTGGTGCCCATCCTG
SFHR047	SBmGW-K6 as	ATGGGCACCACGGTGAACAGTTCCTCGCCCTTGGACACCATGGTT
SFHR048	SBmGW-K16 s	ATGGTGTCCAAGGGCGAGGAACTGTTCACCGCGAGCGTGGTGCCCATCCTG
SFHR049	SBmGW-K16 as	ATGGGCACCACGCTCGCGGTGAACAGTTCCTCGCCCTTGGACACCATGGTT
SFHR050	SBmGW-K18 s	ATGGTGTCCAAGGGCGAGGAACTGTTCACCGCGGGCGTGGTGCCCATCCTG
SFHR051	SBmGW-K18 as	ATGGGCACCACGCCCGCGGTGAACAGTTCCTCGCCCTTGGACACCATGGTT
SFHR052	SBmGW-K29 s	ATGGTGTCCAAGGGCGAGGAACTGTTCACCGCGGTGCCCATCCTG
SFHR053	SBmGW-K29 as	ATGGGCACCGCGGTGAACAGTTCCTCGCCCTTGGACACCATGGTT
SFHR054	SBmGW-K33 s	ATGGTGTCCAAGGGCGAGGAACTGTTCACCGGCGCGGTGCCCATCCTG
SFHR055	SBmGW-K33 as	ATGGGCACCGCGCCGGTGAACAGTTCCTCGCCCTTGGACACCATGGGT
**Generation of CYBB mutant vectors**		
BH111	SB173CW s	GGGCGGCCTGTACCTGGCTGTGACCTGTTGGCCGGCATCACCGGCGTG
BH112	SB173CW as	CACGCCGGTGATGCCGGCCAACAGGTCACAGCCAGGTACAGGCCGCCC
BH113	SB54CW s	GCTGCTGGGCTCAGCACTGGCACTGGCCCAGGGCCCCTGCCGCCTGCCTGAA
BH114	SB54CW as	TTCAGGCAGGCGGCAGGGGCCCTGGGCCAGTGCCAGTGCTGAGCCCAGCAGC
BH121	SB124CW s	GCGCCATCCACACCATCGCACATCTATTTAATGTGTAATGGTGCGTGAACGCCCGGGTG
BH122	SB124CW as	CACCCGGGCGTTCACGCACCATTACACATTAAATAGATGTGCGATGGTGTGGATGGCGC
BH123	SB45CW s	CGACATCCCCCCCAAGTTCTTTTACACAAGAAAACGTCTTGGCAGCGCCCTGGCCCTGG
BH124	SB45CW as	CCAGGGCCAGGGCGCTGCCAAGACGTTTTCTTGTGTAAAAGAACTTGGGGGGGATGTCG
**Shotgun cloning**		
AR36	Shotg HPRT s	TCCCTTCATAGAGACAAGGAATG
AR163	Shotg HPRT as	CCTGGGTTCTACCCCAGCACAG
AR102	Shotg EGFP as	TCATGTGGTCGGGGTATCTG
SFHR20	Shotg EGFP as	CTTGTACAGCTCGTCCATGC
SFHR43	Shotg IRES s	TCTGTAGCGACCCTTTGCAG
BH234	Shotg CYBB as	AGGATCAGGCACAGGGTGAT
**Surveyor assay**		
BH229	Svyr45/54 s	TGGCTCTCCTCAAGCGTATT
BH230	Svyr45/54 as	CCATCTTGTGGAAGGTCAGG
BH231	Svyr173 s	CACCTGTTCAACGTGGAGTG
BH232	Svyr173 as	CTCGCACAGGTACAGGAACA
BH233	Svyr124 s	AACGTGTTCCTGTTCGTGTG
BH234	Svyr124 as	AGGATCAGGCACAGGGTGAT
SFHR 135	Svyr54_off1 s	ATTCCTTTCAACGCTAGCCTTC
SFHR 136	Svyr54_off1 as	GTACATTTGTTTGCCATCACCG
SFHR 137	Svyr54_off2 s	AAGCCATGGTCCAAAATCCTG
SFHR 138	Svyr54_off2 as	CCTGTTGTTCACAGCTCTATGT

SFHR141	Svyr54_off3 s	TGCAGGATCTGGTCCCTAATC
SFHR142	Svyr54_off3 as	GGAATCTCCCATCCTAGCCTAG
SFHR143	Svyr54_off4 s	CGGTCGTTGATTTCTGAGGAG
SFHR144	Svyr54_off5 as	ACAGAGCCTTGGTCTCATGA
SFHR145	Svyr173_off1 s	ACCCACTACGCACATATATATGC
SFHR146	Svyr173_off1 as	TAATGACCTGCAAACAAACCTAC
SFHR147	Svyr173_off2 s	CTCTGTGTTCAGCTCCAGC
SFHR148	Svyr173_off2 as	GTGATTCACAAACAAGGAGGTG
SFHR151	Svyr173_off3 s	CTTTGGCTCTGATGTTATATCTGTAG
SFHR152	Svyr173_off3 as	TCATCAGCATATCGTCTTGCC
SFHR153	Svyr173_off4 s	ACTTCTGCTTCAGGAATTGCC
SFHR154	Svyr173_off4 as	CTACTGGTGCCAGCAAAGAC

Mutation	DNA change	Protein Alteration	Accesion Number	sgRNA Sequence
SB*54*CW	c.159dupC	p.R54fs	A53X102	CAGCACTGGCACTGG**C**CCA**GGG**
SB*173*CW	c.517delC	p.L173fs	L173X188	GTACCTGGCTGTGACC**-**TGT**TGG**
SB*124*CW	c.370G>T	p.E124X	E124X	ACATCTATTTAATGTG**T**AA**TGG**
SB*45*CW	c.134T>G	p.L45R	L45R	TTTACACAAGAAAAC**G**TCT**TGG**

Supplemental Table 2: Selected *CYBB* disease mutations and nucleotide sequences of the *CYBB* targeting sgRNAs.
