# Supplementary material for: High Efficiency Gene Correction in Hematopoietic Cells by Donor-Template-Free CRISPR/Cas9 Genome Editing
Source: Mol Ther Nucleic Acids. 2017 Nov 10;10:1–8. doi: 10.1016/j.omtn.2017.11.001 (PMC5723376; doi:10.1016/j.omtn.2017.11.001)
Supplement: Document S2. Article plus Supplemental Information [file mmc2.pdf]

Molecular Therapy
Nucleic Acids
Original Article

AMERICAN SOCIETY of GENE & CELL THERAPY

High Efficiency Gene Correction in Hematopoietic Cells by Donor-Template-Free CRISPR/Cas9 Genome Editing

Duran Sürün,[1,2] Joachim Schwäble,[2,3] Ana Tomasovic,[1] Roy Ehling,[1,2] Stefan Stein,[4] Nina Kurrle,[1] Harald von Melchner,[1,2] and Frank Schnütgen[1,2]

[1]Department of Molecular Hematology, Goethe University Medical School, 60590 Frankfurt am Main, Germany; [2]LOEWE Center for Cell and Gene Therapy, Goethe University Medical School, 60590 Frankfurt am Main, Germany; [3]Institute for Transfusion Medicine und Immunohematology, Goethe University Medical School, 60528 Frankfurt am, Germany; [4]Institute for Tumor Biology and Experimental Therapy, Georg-Speyer-Haus, 60596 Frankfurt am Main, Germany

The CRISPR/Cas9 prokaryotic adaptive immune system and its swift repurposing for genome editing enables modification of any prespecified genomic sequence with unprecedented accuracy and efficiency, including targeted gene repair. We used the CRISPR/Cas9 system for targeted repair of patient-specific point mutations in the Cytochrome b-245 heavy chain gene (*CYBB*), whose inactivation causes chronic granulomatous disease (XCGD)—a life-threatening immunodeficiency disorder characterized by the inability of neutrophils and macrophages to produce microbicidal reactive oxygen species (ROS). We show that frameshift mutations can be effectively repaired in hematopoietic cells by non-integrating lentiviral vectors carrying RNA-guided Cas9 endonucleases (RGNs). Because about 25% of most inherited blood disorders are caused by frameshift mutations, our results suggest that up to a quarter of all patients suffering from monogenic blood disorders could benefit from gene therapy employing personalized, donor template-free RGNs.

INTRODUCTION

The vast majority of inherited monogenic disorders are caused by patient-specific mutations dispersed over the entire locus of the affected gene.[1] Although correcting mutations by introducing healthy gene copies into the genome of diseased cells proved effective in several clinical gene therapy trials,[2] insertional mutagenesis and unregulated transgene expression remain a concern for randomly integrating vectors (reviewed by Naldini[3]).

Ideally, diseased genes would be corrected directly at their endogenous loci by homologous recombination (HR). Although the original technology developed for gene targeting in mouse embryonic stem cells was successfully upscaled for high throughput generation of knockout mice,[4] its efficiency is quite variable and ineffective in human somatic cells. This changed considerably with the development of designer endonucleases capable of inducing DNA double-strand breaks (DSBs) in any pre-specified genomic sequence that are restored either by homology directed repair (HDR) or non-homologous end joining (NHEJ). Whereas HDR uses a donor DNA template and can be exploited to create specific sequence changes, including targeted addition of whole genes, NHEJ repairs DSBs in the absence of a donor template by religating DNA ends—an error prone process associated with random nucleotide insertions or deletions (indels).

Successful correction of human disease mutations in hematopoietic and induced pluripotent stem cells by designer endonucleases has thus far been based exclusively on HDR. Although HDR offers precision, efficiency is low and most editing protocols rely on positive selection to enrich for gene-corrected cells.[5–12] Because DSB repair by NHEJ in mammalian cells significantly exceeds HDR and, more importantly, is the dominant DSB-repair pathway in hematopoietic stem and progenitor cells (HSPCs),[13,14] we exploited NHEJ for gene repair because, in theory, approximately one-third of indels associated with NHEJ should restore the open reading frame (ORF) disrupted by a disease mutation. This could lead to many ORF reconstitutions, of which some, depending on the position and type of the original mutation, should completely or partially recover protein function, as has been shown recently for the dystrophin gene in patients with Duchenne's muscular dystrophy (DMD).[15]

Here, we show that gene-inactivating point mutations introduced into EGFP transgenes expressed in PLB-985 myeloid leukemia cells are effectively repaired by donor template-free RNA-guided CRISPR/Cas9 endonucleases (RGNs) delivered by integrase-defective lentiviruses (IDLVs). Additionally, mutations in the Cytochrome b-245 heavy chain (*CYBB*) gene encoding nicotinamide adenine dinucleotide phosphate (NADPH) oxidase catalytic gp91[phox] subunit causing X-linked chronic granulomatous disease (XCGD; a life-threatening primary immunodeficiency disorder[16]) can be functionally

Received 20 July 2017; accepted 6 November 2017;
https://doi.org/10.1016/j.omtn.2017.11.001.

Correspondence: Duran Sürün, Department of Molecular Hematology, Goethe University Medical School, 60590 Frankfurt am Main, Germany.
E-mail: d.sueruen@med.uni-frankfurt.de
Correspondence: Harald von Melchner, Department of Molecular Hematology, Goethe University Medical School, 60590 Frankfurt am Main, Germany.
E-mail: melchner@em.uni-frankfurt.de

This is an open access article under the CC BY license (http://creativecommons.org/licenses/by/4.0/).

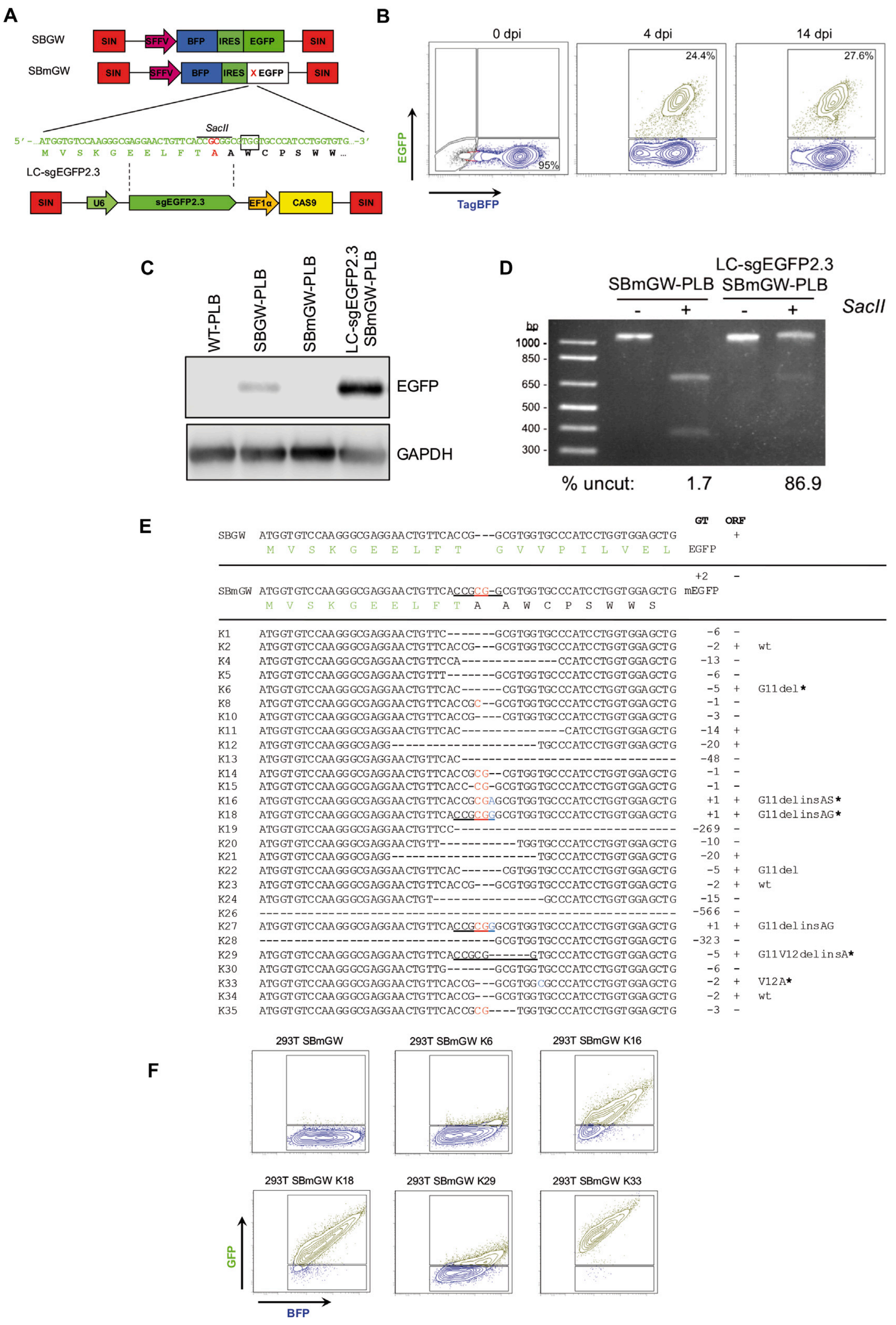

(legend on next page)

reconstituted in *CYBB*-null PLB (XCGD) cells[17] engineered to express patient-specific *CYBB* mutations. With gene repair efficiency of up to 25% for some *CYBB* mutations and an on-target mutation rate of 75% at the endogenous *CYBB* locus, we believe that a donor template-free RGN approach has potential for personalized gene therapy of chronic granulomatous disease (CGD) and other monogenic blood disorders.

RESULTS AND DISCUSSION

To test gene repair efficiency by NHEJ in human hematopoietic cells, we generated PLB-985 (PLB)[18] reporter cells expressing blue fluorescent protein (tagBFP),[19,20] along with either intact (EGFP) or mutationally inactivated EGFP (mEGFP). TagBFP (BFP) was linked to EGFP or mEGFP by an internal ribosomal entry site (IRES), and BFP-IRES-EGFP cassettes were cloned into a self-inactivating (SIN) lentiviral vector downstream of an internal SFFV promoter (Figure 1A). The EGFP mutation consisted of a 2-nt, frameshifting insertion that generated a *SacII* restriction site at the 5′ end of EGFP (Figure 1A). Two lentiviral vectors, SBGW and SBmGW, were used to infect PLB cells (PLBs) at a low multiplicity (MOI 0.01) to obtain single copy integrations (Figure S1). Two days after infection, transduced PLBs were analyzed by fluorescence-activated cell sorting (FACS). As expected, the majority of SBGW-transduced PLBs (SBGW-PLB) were double positive for BFP and EGFP ($BFP^{+}GFP^{+}$), whereas, consistent with EGFP inactivation, SBmGW-transduced PLBs (SBmGW-PLB) expressed only BFP (Figure S2).

Next, we cloned a single guide RNA (sgRNA) targeting the EGFP mutation (sgEGFP2.3, Figure 1A) into the pLentiCRISPRv2 lentiviral vector[21] and infected FACS-sorted BFP^{+}SBmGW-PLBs with IDLVs referred to as LC-sgEGFP2.3. IDLVs were chosen for transient RGN delivery because they can effectively transduce hematopoietic stem cells.[6,22,23] More recently, however, direct RGN delivery by electroporation deemed clinically more compatible was shown to be at least as effective as IDLVs.[12,24] Figure 1B shows infection of BFP^{+}SBmGW-PLBs with LC-sgEGFP2.3 reconstituted EGFP expression in up to 24% of cells within 4 days post-infection. After 14 days, the fraction of $BFP^{+}EGFP^{+}$ cells increased by another 3%, indicating stable EGFP repair (Figure 1B). Furthermore, western blot analysis revealed robust EGFP protein expression (Figure 1C).

To estimate the on-target mutation rate of LC-sgEGFP2.3, we digested genomic EGFP amplification products from the transduced SBmGW-PLBs with *SacII*, separated restriction fragments on agarose gels, and quantified uncleaved DNA by densitometry. Figure 1D shows that up to 87% of EGFP alleles lost the *SacII* restriction site, which is consistent with a high IDLV-transduction rate.

To determine the type of indels leading to EGFP repair, we shot-gun cloned genomic EGFP amplification products from IDLV-transduced SBmGW-PLBs into the pGEM-T vector and isolated 28 bacterial clones after transformation into *E. coli*. pGEM-T insert sequencing revealed that 13 of 28 indels (46%) restored the EGFP-ORF (Figure 1E). Four of these were 2-nt deletions (K2, K23, K33, and K34), of which three restored the wild-type sequence and one (K33) converted a Val codon into Ala. Although a monoclonal origin of the three wild-type indels cannot be excluded, clonal outgrowth was deemed unlikely in the absence of selection. The other ORFs included amino acid substitutions combined with acquisitions induced by 1-nt insertions or amino acid deletions combined with substitutions induced by 5′ nucleotide deletions (Figure 1E). Importantly, none of the recovered sequences contained the original SBmGW mutation, suggesting an on-target mutation rate approaching 100%.

To test whether non-canonical ORFs are compatible with EGFP fluorescence, we replicated recovered ORFs in SBGW vectors by site-specific mutagenesis and individually transfected these into HEK293T cells. After 48 hr, FACS analysis identified K16, K18, and K33 ORFs compatible with EGFP expression because most transfected BFP^{+} cells were also positive for EGFP. In contrast, single amino acid deletions from K6 and K29 ORFs were incompatible with EGFP expression (Figure 1F). This is consistent with previous reporting showing that N-terminal EGFP mutations abolish EGFP fluorescence.[25]

Overall, 25% of the indels repaired mEGFP—a frequency similar to the fraction of $EGFP^{+}$ cells recovered from LC-sgEGFP2.3-transduced SBmGW-PLB cells (Figure 1B).

To test whether the donor template-free RGN-IDLV strategy would also correct *bone fide* disease mutations, we replaced EGFP in the SBGW vector with wild-type or mutated *CYBB* cDNAs. We generated 5 BFP-IRES-CYBB lentiviral vectors carrying either wild-type *CYBB* (SB*wt*CW) or one of the following XCGD patient-specific mutations: frameshift-*R54fs*CYBB (SB*54*CW), frameshift-*L173fs*CYBB (SB*173*CW), nonsense- *E124X*CYBB (SB*124*CW), or missense-*L45R*CYBB (SB*45*CW) (Figure 2A; Table S1).

Wild-type and mutant *CYBB*-carrying lentiviruses were transduced into *CYBB*-null PLB cells (XCGD cells)[17] by low MOI infection and

Figure 1. EGFP Repair Efficiency in PLB Cells Expressing Dual Color Reporters

(A) Lentiviral reporter constructs with cDNAs encoding blue fluorescent protein (tag BFP) and either wild-type (SBGW) or mutated (SBmGW) EGFP (top) and schematic representation of the LC-sgEGFP2.3 lentiviral vector with its target sequence (bottom). (B) Frequency of $EGFP^{+}$ cells among FACS-sorted BFP^{+} SBmGW PLB cells before and after LC-sgEGFP2.3 IDLV infection (MOI 11). (C) Western blot showing EGFP expression in WT, unsorted SBGW-PBL control cells and in sorted BFP^{+} SBmGW-PLB cells before and after IDLV infection. (D) *SacII* digests of genomic EGFP amplification products from SBmGW-PLB cells before and after IDLV treatment. Numbers at the bottom represent the amount of uncut DNA estimated by densitometry. (E) Indel sequences recovered by shot-gun cloning. Reconstituted *SacII* restriction sites are underlined. (F) FACS analysis of HEK293T cells expressing mEGFP cDNAs reconstituted by non-canonical ORFs. For further explanation, see text. BFP, blue fluorescent protein; EF1a, elongation factor 1 alpha short variant promoter; EGFP, enhanced green fluorescent protein; IRES, internal ribosomal entry site; pA, polyadenylation site; SFFV, spleen focus forming virus promoter; SIN, self-inactivating long terminal repeat (LTR); U6, human RNA polymerase III promoter.

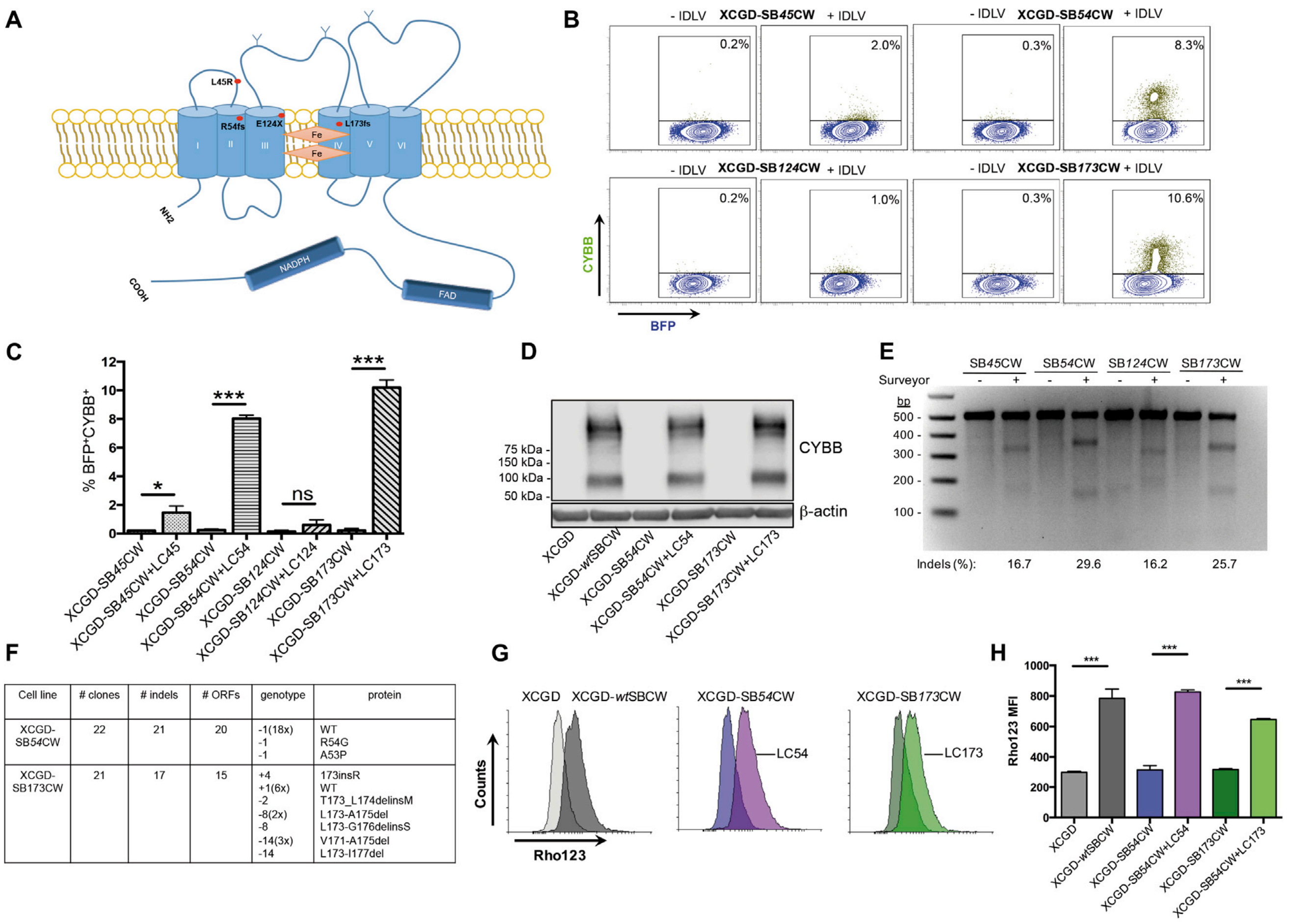

Cell line	# clones	# indels	# ORFs	genotype	protein
XCGD-SB*54*CW	22	21	20	-1(18x)<br>-1<br>-1	WT<br>R54G<br>A53P
XCGD-SB173CW	21	17	15	+4<br>+1(6x)<br>-2<br>-8(2x)<br>-8<br>-14(3x)<br>-14	173insR<br>WT<br>T173_L174delinsM<br>L173-A175del<br>L173-G176delinsS<br>V171-A175del<br>L173-I177del

Figure 2. Repair of *CYBB* Mutations in XCGD-PLB Cells

(A) Schematic representation of CYBB and positions of the selected disease mutations. (B) FACS profiles of sorted BFP+ XCGD cells stained with monoclonal 7D5 anti-human CYBB antibody 4 days after RGN transduction. (C) Frequency of CYBB+ cells among BFP+ XCGD cells. Results are represented as mean ± SD of 3 independent experiments. (D) Western blot showing CYBB expression in IDLV-transduced BFP+ XCGD cells harboring the different CYBB mutations. (E) Surveyor assay performed with *CYBB* PCR products of IDLV-treated XCGD cells. Indel frequency was calculated according to the formula published by Hsu et al.[39] (F) Types of indels recovered from FACS-sorted CYBB+ XCGD cells. (G) Representative histograms depicting ROS production by differentiated, CYBB+ XCGD cells after stimulation with PMA. ROS levels were estimated by measuring the oxidative conversion of dihydrorhodamine 123 into rhodamine 123, which exhibits green fluorescence (DHR assay). (H) Mean fluorescence intensity induced by differentiated XCGD cells before and after IDLV transduction. XCGD and XCGD-SB*wt*CW cells served as negative and positive controls, respectively. Results are represented as mean ± SEM of 2 to 3 independent experiments. ***$p < 0.001$; *$p < 0.05$.

transduced XCGD cells were analyzed for BFP- and CYBB expression by flow cytometry 4 days post-infection. Most SB*wt*CW-transduced XCGD cells expressed both BFP and CYBB, whereas all XCGD cells carrying mutant copies expressed only BFP (Figure S3).

Next, we cloned sgRNAs targeting different *CYBB* mutations (Table S1) into pLentiCRISPRv2 and infected FACS-sorted, BFP+XCGD cells with corresponding LC-sgCYBB IDLVs. After 14 days, up to 10% of XCGD-*54*CW and XCGD-*173*CW cells stained positive for CYBB (Figures 2B and 2C) and expressed full-length CYBB, as revealed by western blotting (Figure 2D). Although repair efficiency was only half of that achieved for mEGFP in SBmGW-PLB cells (Figure 1B), so was the on-target mutation rate (Figure 2E). In contrast, less than 2% of XCGD-*124*CW and XCGD-SB*45*CW cells stained positive for CYBB (Figures 2B and 2C), suggesting that nonsense and missense mutations are less amenable to RGN repair. However, both mutations also showed reduced on-target mutation rates (Figure 2E), presumably caused by the low CG content of the respective sgRNA (Table S1).[26] Moreover, Cas9 tolerance of single nucleotide mismatches[27] could have selected against single nucleotide substitutions.

To indentify indels compatible with CYBB expression, we sequenced several indels from FACS-sorted CYBB+, XCGD-*54*CW, and XCGD-*173*CW cells recovered by shot-gun cloning. As anticipated, most indels from XCGD-*54*CW and XCGD-*173*CW mutations reconstituted the ORFs (Figures 2F and S4). For the XCGD-*54*CW mutation, only two

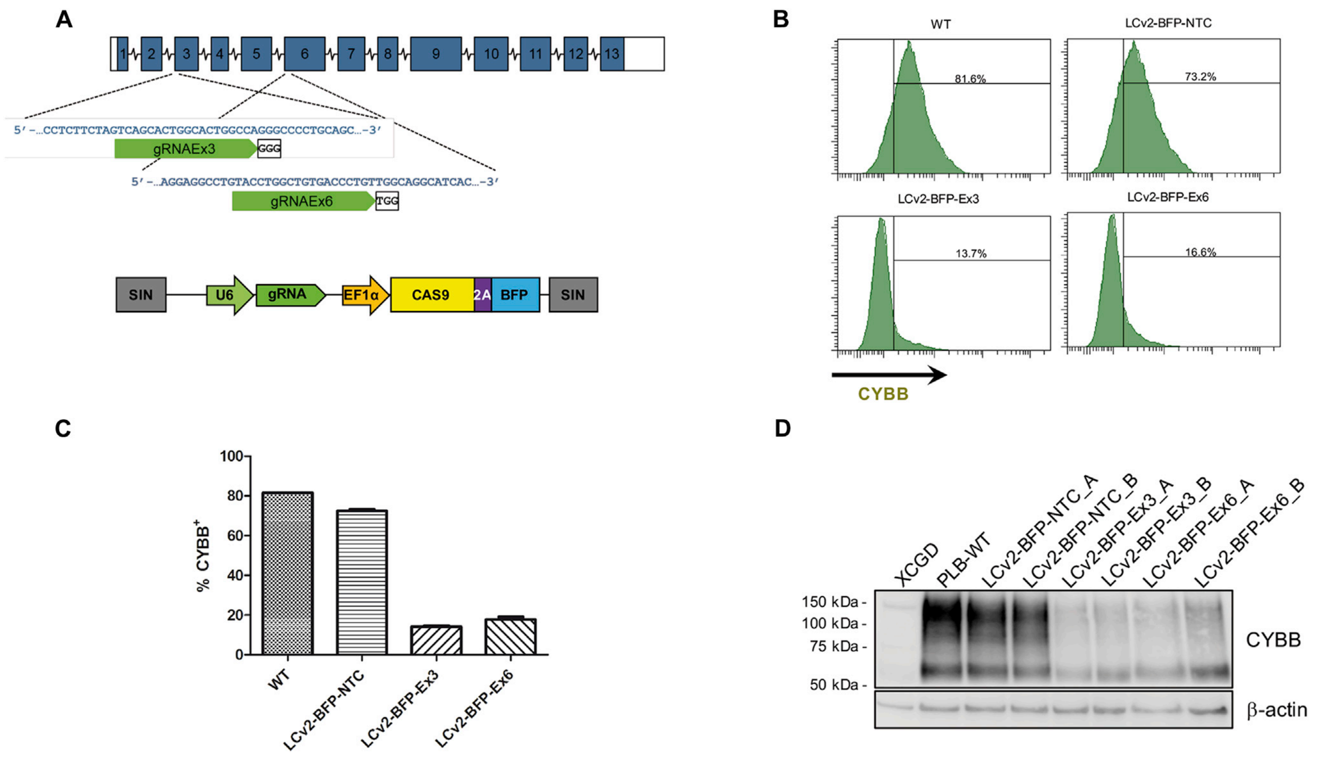

Figure 3. Targeting Efficiency at the Endogenous *CYBB* Locus

(A) Lentiviral construct (bottom) used to target the endogenous CYBB locus (top) with the corresponding sgRNAs. (B) FACS profiles of CYBB-expressing PLB cells 14 days after RGN transduction. (C) Bar graph showing the frequency of $CYBB^+$ cells after RGN transduction. Results are represented as mean ± SEM of 2 independent experiments. (D) Western blot showing reduced CYBB expression in RGN-transduced PLB cells. (A) and (B) denote independent experiments. Ex, exon; NTC, non-target control; WT, untreated control.

ORFs with single nucleotide deletions failed to match the wild-type sequence. Although one of these might be compatible with CYBB expression because it retains Arg^{54} (K21, Figure S4), which is essential for reactive oxygen species (ROS) production, the other Arg54Gly mutation is likely non-functional.[28] In contrast, over half of the ORFs reconstituting the XCGD*173*CW mutation residing in the 4th transmembrane domain of CYBB (Figure 2A) were non-canonical, including one 4-nt insertion, one 2-nt deletion, three 8-nt deletions, and four 14-nt deletions (Figures 3F and S4). According to SMART, the *in silico* modular architecture research tool (http://smart.embl-heidelberg.de/), none of these ORFs appears to affect the integrity of the transmembrane domain, explaining why gene repair efficiency was highest in XCGD-*173*CW cells (Figures 2B and 2C).

Next, we PCR amplified 400–500 bp genomic DNA fragments from the top 4 off-target loci in IDLV-treated XCGD-54CW and XCGD-173CW cells (Figure S5A) and subjected these to the Surveyor assay. Consistent with a high sgRNA specificity, no cleavage was found in the off-targets of both RGN-transduced cell lines (Figure S5B).

To test whether CYBB expression is equivalent to gene repair, we estimated ROS production by differentiated $CYBB^+$, XCGD-*54*CW, and XCGD-*173*CW cells using the dihydrorhodamine-123 (DHR) reduction assay.[29] Figures 3B and 3G show *CYBB*-corrected XCGD-*54*CW and XCGD-*173*CW neutrophils obtained after DMSO induced differentiation (Figure S6), which produced superoxide equivalent to wild-type CYBB expressing (XCGD-SB*wt*CW) cells.

Finally, to determine whether the NHEJ gene repair strategy would have similar efficiency at the endogenous *CYBB* locus, we transduced wild-type PLB cells with LC-BFP-sgCYBB LVs, in which sgRNAs targeting R54fs and L173fs mutations were replaced with sgRNAs targeting corresponding wild-type sequences (sgEx3 and sgEx6; Figure 3A; Table S1). Non-transduced PLB cells and PLB cells transduced with LVs expressing scrambled (off-target) sgRNA (LC-NTC; Table S1) served as positive controls. Flow cytometry 4 days post-transduction, showed that about 60% of LC-sgEx3- and LC-sgEx6-LV transduced cells ceased to express CYBB (Figure 3B), which was confirmed by western blotting (Figure 3C). To directly estimate the on-target mutation rate, we sequenced several indels recovered by shot-gun cloning from LC-sgEx3- and LC-sgEx6-LV transduced cells. As shown in Figure S7, 21 out of 25 exons 3 and 19 out of 25 exons 6 exhibited a mutation, suggesting an on-target mutation rate of over 75%. Assuming gene repair efficiencies of about 25%, we predict an *in situ* gene repair efficiency of at least 18%, which is sufficient to protect X-CGD patients from microbial infections.[30,31]

Although these observations require validation with patient material, they nevertheless demonstrate that frameshift mutations can be effectively repaired in hematopoietic cells by donor-template free CRISPR/Cas9 technology. According to the CYBBbase database (http://structure.bmc.lu.se/idbase/CYBBbase/browser.php?content=browser), 24% of XCGD patients harbor *CYBB* frameshift mutations. Because this frequency is similar to the frequency of frameshift mutations recovered in the *IL2Rγ*[32] (http://www.ncbi.nlm.nih.gov/lovd/home.php?select_db=IL2RG), *WASP*[33] (http://pidj.rcai.riken.jp/waspbase/), *ADA* (http://structure.bmc.lu.se/idbase/ADAbase/index.php?content=pubs/Idbases) and *HBB*[34] (http://globin.cse.psu.edu/globin/hbvar/) genes of patients with X-linked immunodeficiency disease (X-SCID), Wiskott-Aldrich Syndrome, adenosinedeaminase immunodeficiency disease (ADA-SCID), and β-thalassemia, respectively, one in four of these patients is likely to benefit from gene therapy with donor template-free, RNA-guided Cas9 endonucleases.

MATERIALS AND METHODS

Vectors and Endonucleases

Cas9 and gRNA_Cloning vector used for the nucleofection experiments were purchased from Addgene (plasmids #41815 and #41824).[35] The gRNA_Cloning vector was optimized for gRNA cloning and expression by inserting the partially missing U6 promoter and gRNA scaffold sequences into the *SpeI* and *NdeI* sites of the gRNA_Cloning vector.[35] The EGFP-targeting sgRNAs obtained by annealing the BH001–BH004 and BH037–BH044 oligonucleotides (Table S1) were cloned into the modified gRNA_Cloning vector using the Golden Gate Protocol.[36]

The dicistronic BFP/EGFP and BFP/CYBB lentiviral vectors were generated by first inserting EGFP- and CYBB cDNAs into *MscI* and *SbfI* sites of the TagBFP-expressing lentiviral vector -pHR'SINcPPT-SBW obtained from M. Grez.[37] Subsequently, an ECMV-IRES was cloned into the *MscI* site between the BFP/EGFP or BFP/CYBB cDNAs.

EGFP mutations were introduced by inserting annealed synthetic oligonucleotides into the BstXI sites of EGFP (Oligos SFHR037, SFHR038, and SFHR046–SFHR055). CYBB mutations were introduced by standard site-specific mutagenesis using the BH137–BH144 primers (Table S1).

pLentiCRISPRv2 vectors containing the different sgRNAs were obtained by target-specific oligonucleotide annealing (Table S1) using the GoldenGate protocol.

Cell Culture

Hematopoietic cells were grown in RPMI-1640 medium supplemented with 10% (v/v) heat-inactivated fetal calf serum (FCS), 2 mM glutamine, 100 U/mL penicillin, and 100 μg/mL streptomycin.

Lentiviral Vector Production

Cell-free, lentiviral supernatants were produced by polyethylenimine (PEI) based transient co-transfection of HEK293T cells. Briefly, pLentiCRISPRv2 vectors, lentiviral *gag/pol* helper plasmids for integrating (pCMV8.91) or integrase defective (pCMV8.74) variants and envelope plasmid encoding the glycoprotein of vesicular stomatitis virus (VSV-G) (pMD2.G, Addgene #12259) were transfected at a molar ratio of 3:1:1 by standard PEI transfection. 48 hr post transfection, viral supernatants were harvested, sterile filtered (0.45-μm pore-size PVDF-membrane filter; Millipore, Schwalbach, Germany), and concentrated (60- to 100-fold) by ultracentrifugation over a 20% (w/v) sucrose cushion (50,000 $\times$ *g*, 2 hr, 4°C). Pelleted vector particles were resuspended in StemSpan SFEM serum-free medium (STEMCELL Technologies, Grenoble, France) without any supplements and stored at −80°C. SBGW lentiviral titers were determined in serial dilutions of viral supernatant by transduction of PLB-985 cells, followed by flow cytometry 4 to 5 days post-transduction. For determining IDLV titers, p24 viral coat protein concentrations were estimated in the viral supernatants using the p24 ELISA kit from INNOTEST (Fujirebio, Hannover, Germany) according to the manufacturer's instructions. To determine the number of particles corresponding to every picogram of p24 antigen, we used a conversion factor of 6.12×10^3 particles/pg derived from the flow cytometric analysis of PLB cells 48 hr after SBmGW-IDLV transduction.

Flow Cytometry and Cell Sorting

For flow cytometry, cells were washed and resuspended in PBS. For cell surface antigen staining, cells were incubated in the dark with fluorescein isothiocyanate (FITC)-conjugated anti-flavocytochrome b558 7D5 (D162-4) (MBL International, Woburn, MA, USA) or CD11b-APC (Miltenyi Biotec no. 130-098-088) antibodies for 20–30 minutes at room temperature. Data acquisition was performed with a BD LSRFortessa flow cytometer (BD Biosciences, Heidelberg, Germany). Data were analyzed with BD FACSDiva software (BD Biosciences, Heidelberg, Germany) or flowing software 2.5.1. Cell sorting was performed in a BD FACSAria III flow cytometer (BD Biosciences, Heidelberg, Germany).

Molecular Analyses

For indel identification, PCR products were generated using primers SFHR020/SFHR043 for exogenous EGFP, BH229/BH234 for exogenous CYBB, SFHR128/SFHR129 for endogenous Ex3, and SFHR132/SFHR133 for endogenous Ex6 (Table S1). Shot gun cloning of PCR amplification products was performed using the pGEM-T vector system (Promega, Mannheim, Germany) according to the manufacturer's instructions. pGEM-T inserts derived from individual bacterial colonies were sequenced using the standard M13-forward primer.

Western Blot

Cells were lysed for 30 min on ice in lysis buffer (50 mM Tris, pH 7.4, 0.15 M NaCl, 2 mM EDTA, and 1% NP-40) supplemented with Protease Inhibitor Cocktail (Roche, Mannheim, Germany). The samples were resuspended in loading buffer containing 20% beta-mercaptoethanol, boiled 5 min at 95°C, and separated by SDS-PAGE. Mouse monoclonal antibody against GFP was purchased from Roche, and rabbit monoclonal antibody against GAPDH was purchased from

Cell Signaling (Frankfurt am Main, Germany). Monoclonal anti-human CYBB antibody (moAB48) was obtained from LifeSpan BioSciences (Seattle, WA, USA).

Surveyor Assay

RGN-targeted CYBB sites were PCR amplified to obtain 500-nt-sized products using the BH229/BH230, BH231/BH232, and BH233/BH234 primers (Table S1). For the analysis of on-target mutation rates, 400 ng PCR product was subjected to the Surveyor assay using the Surveyor Mutation Detection Kit-S100 (IDT, Leuven, Belgium) and the manufacturer's instructions. Indel percentages were derived from ImageJ plots[38] using the following formula: $100 \times (1 - (1 - (b + c)/(a + b + c))^{0.5})$, where a is the integrated intensity of the undigested PCR product, and b and c are the integrated intensities of each cleavage product.

DHR Reduction Assay

DHR assays were performed as described by Brendel et al.[29] Briefly, for granulocytic differentiation, cells were plated at a concentration of 2×10^5 cells/mL in RPMI-1640 supplemented with 2.5% heat inactivated FCS, 2 mM glutamine, 100 U/mL penicillin, and 100 μg/mL streptomycin and 1.25% dimethyl sulfoxide (Sigma-Aldrich, Taufkirchen, Germany) for at least 7 days. For estimating ROS production, the differentiated cells were suspended in 1 mL pre-warmed Hank's balanced salt solution (HBSS) (Life Technologies, Darmstadt, Germany), supplemented with 7.5 mmol/L D-Glucose, 0.5% bovine serum albumin (BSA) (Sigma-Aldrich), 2,000 U/mL catalase (Sigma-Aldrich), and 5 μg/mL DHR123 (Sigma-Aldrich). Following incubation for 10 min at 37°C, cells were exposed to 0.1 μmol/L phorbol 12-myristate 13-acetate (PMA; Sigma-Aldrich) for 15 min and placed on ice. Rhodamin 123 fluorescence was measured in a flow cytometer within the next 30 min.

Statistical Analysis

For statistical comparisons between groups, Student's t test or one-way ANOVA with Bonferoni post hoc test were used as appropriate in conjunction with GraphPad Prism 5 software.

SUPPLEMENTAL INFORMATION

Supplemental Information includes seven figures and two tables and can be found with this article online at https://doi.org/10.1016/j.omtn.2017.11.001.

AUTHOR CONTRIBUTIONS

D.S., J.S., A.T., R.E., S.S., N.K., and F.S. conducted the experiments. D.S., H.v.M., and F.S. conceived the study, designed the experiments, and wrote the paper.

ACKNOWLEDGMENTS

We thank Nadine Happel for expert technical assistance. This work was supported by individual grants from the Deutsche Forschungsgemeinschaft to H.v.M. (ME 820/6-1) and F.S. (SCHN1166/4-1). Further support was provided by the Bundesministerium für Bildung und Forschung (NGFNplus-DiGtoPconsortium/01GS0858) to H.v.M. as well as an individual grant from the Loewe Center for Cell and Gene Therapy to F.S.

REFERENCES

1. Pessach, I.M., and Notarangelo, L.D. (2011). Gene therapy for primary immunodeficiencies: looking ahead, toward gene correction. J. Allergy Clin. Immunol. *127*, 1344–1350.
2. Cicalese, M.P., and Aiuti, A. (2015). Clinical applications of gene therapy for primary immunodeficiencies. Hum. Gene Ther. *26*, 210–219.
3. Naldini, L. (2011). Ex vivo gene transfer and correction for cell-based therapies. Nat. Rev. Genet. *12*, 301–315.
4. Skarnes, W.C., Rosen, B., West, A.P., Koutsourakis, M., Bushell, W., Iyer, V., Mujica, A.O., Thomas, M., Harrow, J., Cox, T., et al. (2011). A conditional knockout resource for the genome-wide study of mouse gene function. Nature *474*, 337–342.
5. Urnov, F.D., Miller, J.C., Lee, Y.L., Beausejour, C.M., Rock, J.M., Augustus, S., Jamieson, A.C., Porteus, M.H., Gregory, P.D., and Holmes, M.C. (2005). Highly efficient endogenous human gene correction using designed zinc-finger nucleases. Nature *435*, 646–651.
6. Genovese, P., Schiroli, G., Escobar, G., Tomaso, T.D., Firrito, C., Calabria, A., Moi, D., Mazzieri, R., Bonini, C., Holmes, M.C., et al. (2014). Targeted genome editing in human repopulating haematopoietic stem cells. Nature *510*, 235–240.
7. Sebastiano, V., Maeder, M.L., Angstman, J.F., Haddad, B., Khayter, C., Yeo, D.T., Goodwin, M.J., Hawkins, J.S., Ramirez, C.L., Batista, L.F., et al. (2011). In situ genetic correction of the sickle cell anemia mutation in human induced pluripotent stem cells using engineered zinc finger nucleases. Stem Cells *29*, 1717–1726.
8. Sun, N., and Zhao, H. (2014). Seamless correction of the sickle cell disease mutation of the HBB gene in human induced pluripotent stem cells using TALENs. Biotechnol. Bioeng. *111*, 1048–1053.
9. Ma, N., Liao, B., Zhang, H., Wang, L., Shan, Y., Xue, Y., Huang, K., Chen, S., Zhou, X., Chen, Y., et al. (2013). Transcription activator-like effector nuclease (TALEN)-mediated gene correction in integration-free β-thalassemia induced pluripotent stem cells. J. Biol. Chem. *288*, 34671–34679.
10. Xie, F., Ye, L., Chang, J.C., Beyer, A.I., Wang, J., Muench, M.O., and Kan, Y.W. (2014). Seamless gene correction of β-thalassemia mutations in patient-specific iPSCs using CRISPR/Cas9 and piggyBac. Genome Res. *24*, 1526–1533.
11. Flynn, R., Grundmann, A., Renz, P., Hänseler, W., James, W.S., Cowley, S.A., and Moore, M.D. (2015). CRISPR-mediated genotypic and phenotypic correction of a chronic granulomatous disease mutation in human iPS cells. Exp. Hematol. *43*, 838–848.e3.
12. De Ravin, S.S., Li, L., Wu, X., Choi, U., Allen, C., Koontz, S., Lee, J., Theobald-Whiting, N., Chu, J., Garofalo, M., et al. (2017). CRISPR-Cas9 gene repair of hematopoietic stem cells from patients with X-linked chronic granulomatous disease. Sci. Transl. Med. *9*.
13. Mao, Z., Bozzella, M., Seluanov, A., and Gorbunova, V. (2008). Comparison of nonhomologous end joining and homologous recombination in human cells. DNA Repair (Amst.) *7*, 1765–1771.
14. Beerman, I., Seita, J., Inlay, M.A., Weissman, I.L., and Rossi, D.J. (2014). Quiescent hematopoietic stem cells accumulate DNA damage during aging that is repaired upon entry into cell cycle. Cell Stem Cell *15*, 37–50.
15. Ousterout, D.G., Perez-Pinera, P., Thakore, P.I., Kabadi, A.M., Brown, M.T., Qin, X., Fedrigo, O., Mouly, V., Tremblay, J.P., and Gersbach, C.A. (2013). Reading frame correction by targeted genome editing restores dystrophin expression in cells from Duchenne muscular dystrophy patients. Mol. Ther. *21*, 1718–1726.
16. Heyworth, P.G., Cross, A.R., and Curnutte, J.T. (2003). Chronic granulomatous disease. Curr. Opin. Immunol. *15*, 578–584.
17. Zhen, L., King, A.A., Xiao, Y., Chanock, S.J., Orkin, S.H., and Dinauer, M.C. (1993). Gene targeting of X chromosome-linked chronic granulomatous disease locus in a human myeloid leukemia cell line and rescue by expression of recombinant gp91phox. Proc. Natl. Acad. Sci. USA *90*, 9832–9836.
18. Tucker, K.A., Lilly, M.B., Heck, L., Jr., and Rado, T.A. (1987). Characterization of a new human diploid myeloid leukemia cell line (PLB-985) with granulocytic and monocytic differentiating capacity. Blood *70*, 372–378.

19. Merzlyak, E.M., Goedhart, J., Shcherbo, D., Bulina, M.E., Shcheglov, A.S., Fradkov, A.F., Gaintzeva, A., Lukyanov, K.A., Lukyanov, S., Gadella, T.W., et al. (2007). Bright monomeric red fluorescent protein with an extended fluorescence lifetime. Nat. Methods *4*, 555–557.

20. Subach, O.M., Gundorov, I.S., Yoshimura, M., Subach, F.V., Zhang, J., Grüenwald, D., Souslova, E.A., Chudakov, D.M., and Verkhusha, V.V. (2008). Conversion of red fluorescent protein into a bright blue probe. Chem. Biol. *15*, 1116–1124.

21. Sanjana, N.E., Shalem, O., and Zhang, F. (2014). Improved vectors and genome-wide libraries for CRISPR screening. Nat. Methods *11*, 783–784.

22. Hoban, M.D., Cost, G.J., Mendel, M.C., Romero, Z., Kaufman, M.L., Joglekar, A.V., Ho, M., Lumaquin, D., Gray, D., Lill, G.R., et al. (2015). Correction of the sickle cell disease mutation in human hematopoietic stem/progenitor cells. Blood *125*, 2597–2604.

23. Joglekar, A.V., Hollis, R.P., Kuftinec, G., Senadheera, S., Chan, R., and Kohn, D.B. (2013). Integrase-defective lentiviral vectors as a delivery platform for targeted modification of adenosine deaminase locus. Mol. Ther. *21*, 1705–1717.

24. Hendel, A., Kildebeck, E.J., Fine, E.J., Clark, J., Punjya, N., Sebastiano, V., Bao, G., and Porteus, M.H. (2014). Quantifying genome-editing outcomes at endogenous loci with SMRT sequencing. Cell Rep. *7*, 293–305.

25. Saeger, J., Hytönen, V.P., Klotzsch, E., and Vogel, V. (2012). GFP's mechanical intermediate states. PLoS One *7*, e46962.

26. Wang, T., Wei, J.J., Sabatini, D.M., and Lander, E.S. (2014). Genetic screens in human cells using the CRISPR-Cas9 system. Science *343*, 80–84.

27. Fu, Y., Foden, J.A., Khayter, C., Maeder, M.L., Reyon, D., Joung, J.K., and Sander, J.D. (2013). High-frequency off-target mutagenesis induced by CRISPR-Cas nucleases in human cells. Nat. Biotechnol. *31*, 822–826.

28. Cross, A.R., Heyworth, P.G., Rae, J., and Curnutte, J.T. (1995). A variant X-linked chronic granulomatous disease patient (X91+) with partially functional cytochrome b. J. Biol. Chem. *270*, 8194–8200.

29. Brendel, C., Kaufmann, K.B., Krattenmacher, A., Pahujani, S., and Grez, M. (2014). Generation of X-CGD cells for vector evaluation from healthy donor CD34(+) HSCs by shRNA-mediated knock down of gp91(phox). Mol. Ther. Methods Clin. Dev. *1*, 14037.

30. Holland, S.M. (2010). Chronic granulomatous disease. Clin. Rev. Allergy Immunol. *38*, 3–10.

31. Grez, M., Reichenbach, J., Schwäble, J., Seger, R., Dinauer, M.C., and Thrasher, A.J. (2011). Gene therapy of chronic granulomatous disease: the engraftment dilemma. Mol. Ther. *19*, 28–35.

32. Puck, J.M., Pepper, A.E., Henthorn, P.S., Candotti, F., Isakov, J., Whitwam, T., Conley, M.E., Fischer, R.E., Rosenblatt, H.M., Small, T.N., et al. (1997). Mutation analysis of IL2RG in human X-linked severe combined immunodeficiency. Blood *89*, 1968–1977.

33. Jin, Y., Mazza, C., Christie, J.R., Giliani, S., Fiorini, M., Mella, P., Gandellini, F., Stewart, D.M., Zhu, Q., Nelson, D.L., et al. (2004). Mutations of the Wiskott-Aldrich Syndrome Protein (WASP): hotspots, effect on transcription, and translation and phenotype/genotype correlation. Blood *104*, 4010–4019.

34. Giardine, B., van Baal, S., Kaimakis, P., Riemer, C., Miller, W., Samara, M., Kollia, P., Anagnou, N.P., Chui, D.H., Wajcman, H., et al. (2007). HbVar database of human hemoglobin variants and thalassemia mutations: 2007 update. Hum. Mutat. *28*, 206.

35. Mali, P., Yang, L., Esvelt, K.M., Aach, J., Guell, M., DiCarlo, J.E., Norville, J.E., and Church, G.M. (2013). RNA-guided human genome engineering via Cas9. Science *339*, 823–826.

36. Engler, C., Kandzia, R., and Marillonnet, S. (2008). A one pot, one step, precision cloning method with high throughput capability. PLoS One *3*, e3647.

37. Ward, N.J., Buckley, S.M., Waddington, S.N., Vandendriessche, T., Chuah, M.K., Nathwani, A.C., McIntosh, J., Tuddenham, E.G., Kinnon, C., Thrasher, A.J., et al. (2011). Codon optimization of human factor VIII cDNAs leads to high-level expression. Blood *117*, 798–807.

38. Abramoff, M.D., Magalhães, P.J., and Ram, S.J. (2004). Image processing with ImageJ. Biophoton. Int. *11*, 36–42.

39. Hsu, P.D., Scott, D.A., Weinstein, J.A., Ran, F.A., Konermann, S., Agarwala, V., Li, Y., Fine, E.J., Wu, X., Shalem, O., et al. (2013). DNA targeting specificity of RNA-guided Cas9 nucleases. Nat. Biotechnol. *31*, 827–832.

OMTN, Volume *10*

Supplemental Information

High Efficiency Gene Correction in Hematopoietic Cells by Donor-Template-Free CRISPR/Cas9 Genome Editing

Duran Sürün, Joachim Schwäble, Ana Tomasovic, Roy Ehling, Stefan Stein, Nina Kurrle, Harald von Melchner, and Frank Schnütgen

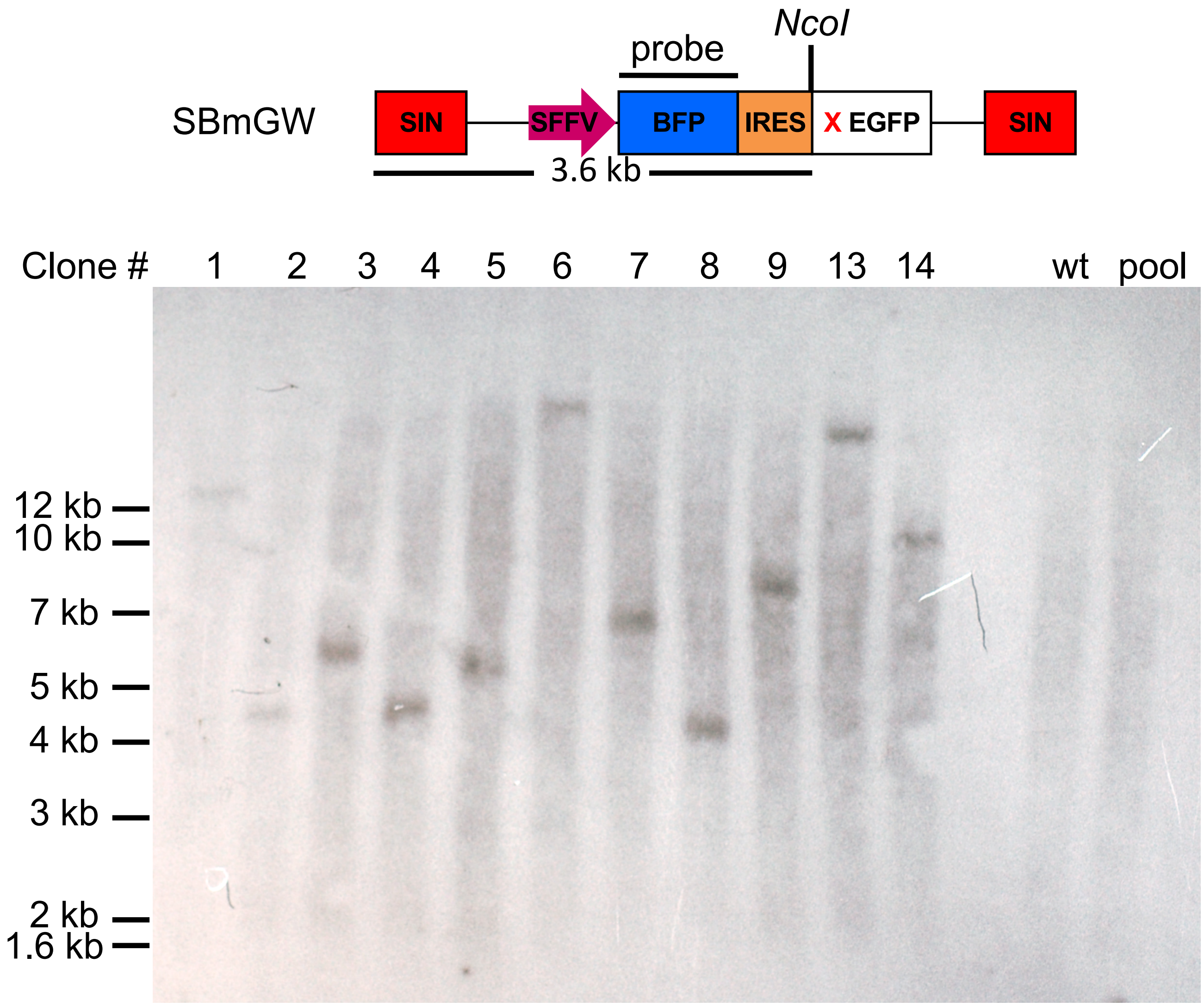

Supplemental Figure 1: Single copy integration of SBmGW lentiviruses in PLB cells. BFP+ SBmGW-PLB clones were isolated by limiting dilution and subjected to Southern blot analysis. *NcoI* restriction fragments of genomic DNAs of individual clones were resolved by agarose gel electrophoresis, transferred onto nylon mebranes and hybridized to a ^{32}P-labeld BFP probe.

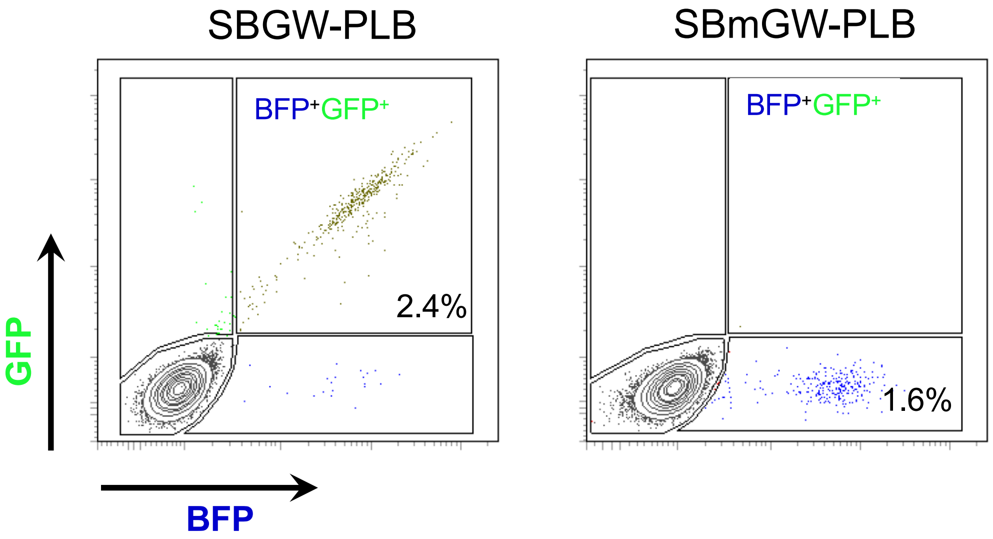

Supplemental Figure 2: FACS profiles of SBGW- and SBmGW-PLB reporter cells

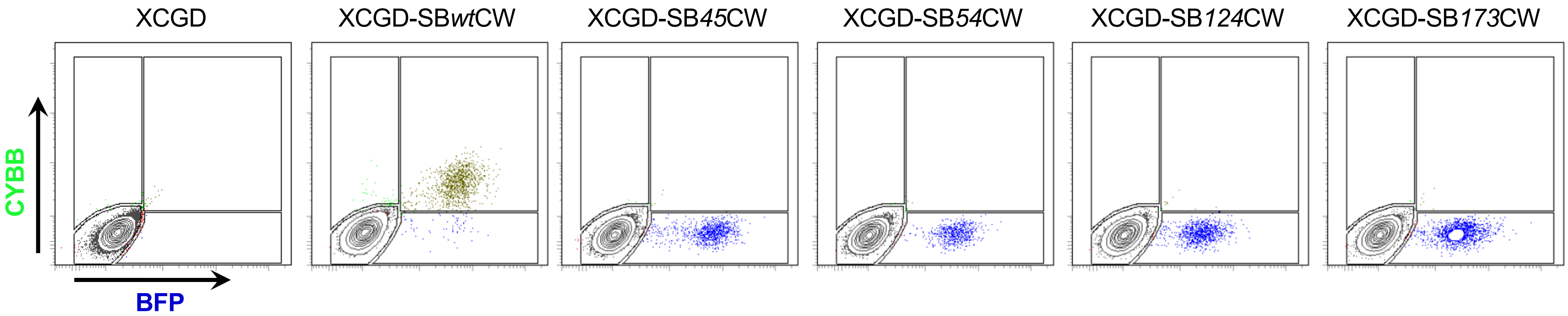

Supplemental Figure 3: FACS profiles of XCGD cells expressing wild-type and mutant CYBB cDNAs. Note that only XCGD-SB*wt*CW cells expressing wild type *CYBB* are double positive for BFP and CYBB.

```
                                                                                   Genotype
SB54CW        CCCGGAAGCTGCTGGGCTCAGCACTGGCACTGGCCCAGGGCCCCTGCCGCCTGCCTGAAC
K1            CCCGGAAGCTGCTGGGCTCAGCACTGGCACTGG-CCAGGGCCCCTGCCGCCTGCCTGAAC -1
K2            CCCGGAAGCTGCTGGGCTCAGCACTGGCACTGG-CCAGGGCCCCTGCCGCCTGCCTGAAC -1
K3            CCCGGAAGCTGCTGGGCTCAGCACTGGCACTGG-CCAGGGCCCCTGCCGCCTGCCTGAAC -1
K4            CCCGGAAGCTGCTGGGCTCAGCACTGGCACTGG-CCAGGGCCCCTGCCGCCTGCCTGAAC -1
K5            CCCGGAAGCTGCTGGGCTCAGCACTGGCACTGGCCCAGGGCCCCTGCCGCCTGCCTGAAC  0
K6            CCCGGAAGCTGCTGGGCTCAGCACTGGCACTGG-CCGGGGCCCCTGCCGCCTGCCTGAAC -1(A>G)
K7            CCCGGAAGCTGCTGGGCTCAGCACTGGCACTGG-CCAGGGCCCCTGCCGCCTGCCTGAAC -1
K8            CCCGGAAGCTGCTGGGCTCAGCACTGGCACTGG-CCAGGGCCCCTGCCGCCTGCCTGAAC -1
K9            CCCGGAAGCTGCTGGGCTCAGCACTGGCACTGG-CCAGGGCCCCTGCCGCCTGCCTGAAC -1
K10           CCCGGAAGCTGCTGGGCTCAGCACTGGCACTGG-CCAGGGCCCCTGCCGCCTGCCTGAAC -1
K11           CCCGGAAGCTGCTGGGCTCAGCACTGGCACTGG-CCAGGGCCCCTGCCGCCTGCCTGAAC -1
K12           CCCGGAAGCTGCTGGGCTCAGCACTGGCACTGG-CCAGGGCCCCTGCCGCCTGCCTGAAC -1
K13           CCCGGAAGCTGCTGGGCTCAGCACTGGCACTGG-CCAGGGCCCCTGCCGCCTGCCTGAAC -1
K14           CCCGGAAGCTGCTGGGCTCAGCACTGGCACTGG-CCAGGGCCCCTGCCGCCTGCCTGAAC -1
K15           CCCGGAAGCTGCTGGGCTCAGCACTGGCACTGG-CCAGGGCCCCTGCCGCCTGCCTGAAC -1
K16           CCCGGAAGCTGCTGGGCTC---------------CCAGGGCCCCTGCCGCCTGCCTGAAC-15
K17           CCCGGAAGCTGCTGGGCTCAGCACTGGCACTGG-CCAGGGCCCCTGCCGCCTGCCTGAAC -1
K18           CCCGGAAGCTGCTGGGCTCAGCACTGGCACTGG-CCAGGGCCCCTGCCGCCTGCCTGAAC -1
K19           CCCGGAAGCTGCTGGGCTCAGCACTGGCACTGG-CCAGGGCCCCTGCCGCCTGCCTGAAC -1
K20           CCCGGAAGCTGCTGGGCTCAGCACTGGCACTGG-CCAGGGCCCCTGCCGCCTGCCTGAAC -1
K21           CCCGGAAGCTGCTGGGCTCAGCACTGGCACTG-CCCAGGGCCCCTGCCGCCTGCCTGAAC -1
K22           CCCGGAAGCTGCTGGGCTCAGCACTGGCACTGG-CCAGGGCCCCTGCCGCCTGCCTGAAC -1
```

```
                                                                                   Genotype
SB173CW       CCCCGAGGGCGGCCTGTACCTGGCTGTGACC----TGTTGGCCGGCATCACCGGCGTGGT
K1            CCCCGAGGGCGGCCTGTACCTGGCTGTGACC----TGTTGGCCGGCATCACCGGCGTGGT   0
K2            CCCCGAGGGCGGCCTGTACCTG------------------GCCGGCATCACCGGCGTGGT -14
K3            CCCCGAGGGCGGCCTGTACCTG------------------GCCGGCATCACCGGCGTGGT -14
K4            CCCCGAAGGCGGCCTGTACCTGGCTGTGA------------CCAGCATCACCGGCGTGGT  -8
K5            CCCCGAGGGCGGCCTGTACCTGGCTGTGA------TGTTGGCCGGCATCACCGGCGTGGT  -6
K6            CCCCGAGGGCGGCCTGTACCTGGCTG------------TGGCCGGCATCACCGGCGTGGT  -8
K7            CCCCGAGGGCGGCCTGTACCTGGCTGTGACCC---TGTTGGCCGGCATCACCGGCGTGGT  +1
K8            CCCCGAGGGCGGCCTGTACCTGGCTGTGACCC---TGTTGGCCGGCATCACCGGCGTGGT  +1
K9            CCCCGAGGGCGGCCTGTACCTGGCTGTGA------------CCGGCATCACCGGCGTGGT  -8
K10           CCCCGAGGGCGGCCTATACCTG-------------TGTTGGCCGGCATCACCGGCGTGGT  -9
K11           CCCCGAGGGCGGCCTGTACCTGGCTGTGACCAGGCTGTTGGCCGGCATCACCGGCGTGGT  +4
K13           CCCCGAGGGCGGCCTGTACCTGGCTGTGACC----TGTTGGCCGGCATCACCGGCGTGGT   0
K14           CCCCGAGGGCGGCCTGTACCTGGCTGTGACCC---TGTTGGCCGGCATCACCGGCGTGGT  +1
K15           CCCCGAGGGCGGCCTGTACCTGGCTGTGACC----TGTTGGCCGGCATCACCGGCGTGGT   0
K16           CCCCGAGGGCGGCCTGTACCTGGCTGTGA------TGTTGGCCGGCATCACCGGCGTGGT  -2
K17           CCCCGAGGGCGGCCTGTACCTGGCTGTGACCC---TGTTGGCCGGCATCACCGGCGTGGT  +1
K18           CCCCGAGGGCGGCCTGTACCTGGCTGTGAC------------------CACCGGCGTGGT -14
K19           CCCCGAGGGCGGCCTGTACCTGGCTGTGACCT---TGTTGGCCGGCATCACCGGCGTGGT  +1
K20           CCCCGAGGGCGGCCTGTACCTG------------------GCCGGCATCACCGGCGTGGT -14
K21           CCCCGAGGGCGGCCTGTACCTGGCTGTGACC----TGTTGGCCGGCATCACCGGCGTGGT   0
K22           CCCCGAGGGCGGCCTGTACCTGGCTGTGACCC---TGTTGGCCGGCATCACCGGCGTGGT  +1
```

Supplementary Figure 4 : Indel sequences recovered by shot gun cloning from XCGD-SB*54*CW and XCGD-SB*173*CW cells.

A

GTACCTGGCTGTGACCTGT**TGG**	sgCYBB54
CAGCACAGACACTGGCCCA**AAG**	chr14:+32749541
CATCACTGGCTCTGGCCCA**TAG**	chr12:-103703290
CAACAGGGGCACTGGCCCA**TGG**	chr1:+204799512
TGGCAGTGGCACTGGCCCA**GGG**	chr19:-46999128
CAGCACTGGCACTGGCCCA**GGG**	sgCYBB173
TTACCTTGCTGTGACCTGT**TAG**	chr2:+186140924
GTTCCTGGCTGTGATCTGT**GAG**	chr7:+73675051
AAACCTAGCTGTGACCTGT**AAG**	chrX:-128220322
CCAACTTGCTGTGACCTGT**GGG**	chr18:+24746359

B

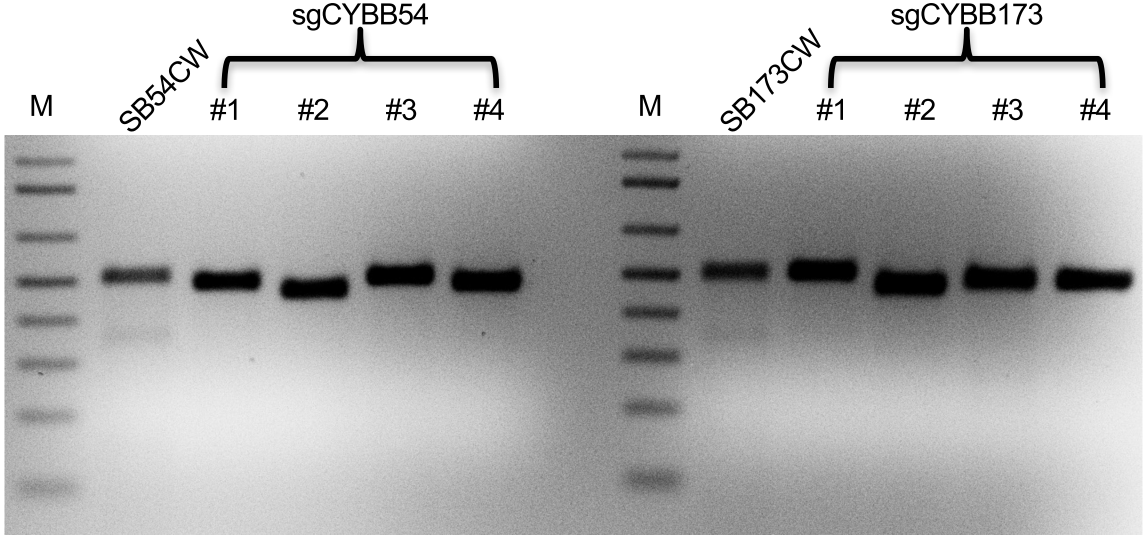

Supplemental Figure 5: Off target analysis in IDLV treated XCGD-54CW and XCGD-173CW cells. **A.** Top 4 off targets for sgCYBB54 and sgCYBB173 RNAs selected from the benchling.com website. **B.** Surveyor assay

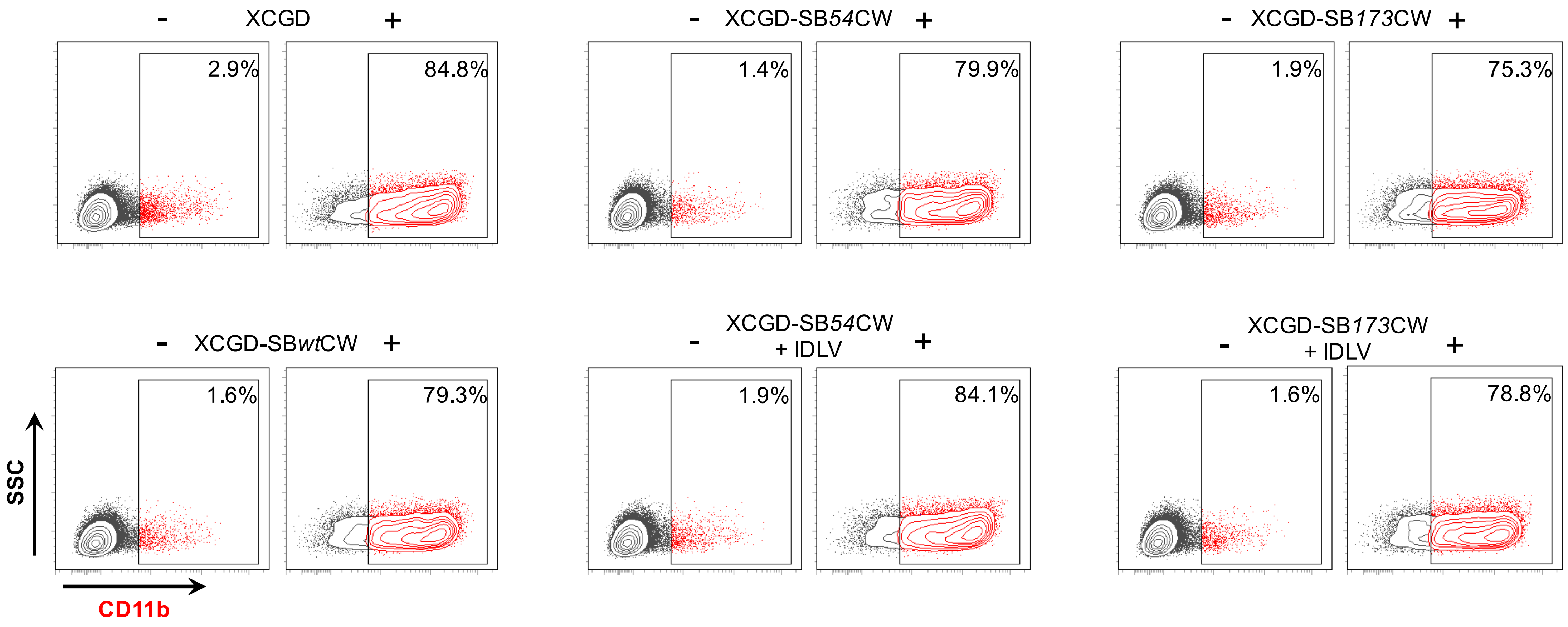

Supplemental Figure 6: Differentiation of XCGD cells after exposure to DMSO. Cells were incubated for 7 days with DMSO (+), stained with anti-CD11b antibody and analyzed by FACS.

```
                                                                                   Genotype

Ex3   CGCCTCTTCTAGTCAGCACTGGCACTGGCCAGGGCCCCTGCAGCCTGCCTGAATTTCAAC
                                                                                      -13
C1    CGCCTCTTCTAGTCAGCACTGGCAC--------------TGCAGCCTGCCTGAATTTCAAC                   -1
C2    CGCCTCTTCTAGTCAGCACTGGCACTG-CCAGGGCCCCTGCAGCCTGCCTGAATTTCAAC                    -1
C3    CGCCTCTTCTAGTCAGCACTGGCACTG-CCAGGGCCCCTGCAGCCTGCCTGAATTTCAAC                    0
C4    CGCCTCTTCTAGTCAGCACTGGCACTGGCCAGGGCCCCTGCAGCCTGCCTGAATTTCAAC                    -1
C5    CGCCTCTTCTAGTCAGCACTGGCACTG-CCAGGGCCCCTGCAGCCTGCCTGAATTTCAAC                    -166
C6    CGCCTCTTCTAGTCAGCACTGGCACTGGCT----------------------------------               -6
C7    CGCCTCTTCTAGTCAGCACTGG------CCAGGGCCCCTGCAGCCTGCCTGAATTTCAAC                    -13
C8    CGCCTCTTCTAGTCAGCACTG--------------CCCCTGCAGCCTGCCTGAATTTCAAC                   -20
C9    CGCCTCTTCTAGTCAGCACTGGCA---------------------CTGCCTGAATTTCAAC                   0
C10   CGCCTCTTCTAGTCAGCACTGGCACTGGCCAGGGCCCCTGCAGCCTGCCTGAATTTCAAC                    -1
C11   CGCCTCTTCTAGTCAGCACTGGCACTG-CCAGGGCCCCTGCAGCCTGCCTGAATTTCAAC                    -2
C12   CGCCTCTTCTAGTCAGCACTGGCACTG--CAGGGCCCCTGCAGCCTGCCTGAATTTCAAC                    -17
C13   CGCCTCTTCTAGTCAGCACTGGCACTGG-----------------TGCCTGAATTTCAAC                    -1
C14   CGCCTCTTCTAGTCAGCACTGGCACTA-CCAGGGCCCCTGCAGCCTGCCTGAATTTCAAC                    -1
C15   CGCCTCTTCTAGTCAGCACTGGCACTG-CCAGGGCCCCTGCAGCCTGCCTGAATTTCAAC                    -120
C16   ----------------------------------------------------------AC                    -1
C17   CGCCTCTTCTAGTCAGCACTGGCACTG-CCAGGGCCCCTGCAGCCTGCCTGAATTTCAAC                    -1
C18   CGCCTCTTCTAGTCAGCACTGGCACTG-CCAGGGCCCCTGCAGCCTGCCTGAATTTCAAC                    -14
C19   CGCCTCTTCTAGTCAGCACTGGCAC--------------TGCAGCCTGCCTGAATTTCAAC                   -1
C20   CGCCTCTTCTAGTCAGCACTGGCACTG-CCAGGGCCCCTGCAGCCTGCCTGAATTTCAAC                    0
C21   CGCCTCTTCTAGTCAGCACTGGCACTGGCCAGGGCCCCTGCAGCCTGCCTGAATTTCAAC                    -2
C22   CGCCTCTTCTAGTCAGCACTGGCACT--CCAGGGCCCCTGCAGCCTGCCTGAATTTCAAC                    0
C23   CGCCTCTTCTAGTCAGCACTGGCACTGGCCAGGGCCCCTGCAGCCTGCCTGAATTTCAAC                    -6
C24   CGCCTCTTCTAGTCAGCACTGGCACTG------GCCCCTGCAGCCTGCCTGAATTTCAAC                    -1
C25   CGCCTCTTCTAGTCAGCACTGGCACTG-CCAGGGCCCCTGCAGCCTGCCTGAATTTCAAC

                                                                                   Genotype

Ex6   CTGAAGGAGGCCTGTACCTGGCTGTGACCCTGTTGGCAGGCATCACTGGAGTTGTCATCA
                                                                                      -26
C1    CTGAAGGAG---------------------------GCAGGCATCACTGGAGTTGTCATCA                   0
C2    CTGAAGGAGGCCTGTACCTGGCTGTGACCCTGTTGGCAGGCATCACTGGAGTTGTCATCA                    -14
C3    CTGAAGGAGGCCTGTACCTGGCTGT--------------GCATCACTGGAGTTGTCATCA                    0
C4    CTGAAGGAGGCCTGTACCTGGCTGTGACCCTGTTGGCAGGCATCACTGGAGTTGTCATCA                    -15
C5    CTGAAGGAGGCCTGTACCTGG---------------CAGGCATCACTGGAGTTGTCATCA                    -1
C6    CTGAAGGAGGCCTGTACCTGGCTGTGACCC-GTTGGCAGGCATCACTGGAGTTGTCATCA                    -47
C7    CTGAAGGAGGCCTGTACCTG----------------------------------------                    -19
C8    CTGAAGGAGGCCTGTACCAG--------------------GCATCACTGGAGTTGTCATCA                   -27
C9    CTGAAGG---------------------------TACAGGCATCACTGGAGTTGTCATCA                    0
C10   CTGAAGGAGGCCTGTACCTGGCTGTGACCCTGTTGGCAGGCATCACTGGAGTTGTCATCA                    0
C11   CTGAAGGAGGCCTGTACCTGGCTGTGACCCTGTTGGCAGGCATCACTGGAGTTGTCATCA                    -1
C12   CTGAAGGAGGCCTGTACCTGGCTGTGACCC-GTTGGCAGGCATCACTGGAGTTGTCATCA                    -10
C13   CTGAAGGAGGCCTGTACCTGT---------GTTGGCAGGCATCACTGGAGTTGTCATCA                     -198
C14   ------------------------------------------------------------                    -16
C15   CTGAAGGAGGCCTGTACCTGG---------------CAGGCATCACTGGAGTTGTCATCA                    -3
C16   CTGAAGGAGGCCTGTACCTGGCTGTGACCC---ATGCAGGCATCACTGGAGTTGTCATCA                    -14
C17   CTGAAGGAGGCCTGTACCTGGCTGTG-------------AATCACTGGAGTTGTCATCA                     -1
C18   CTGAAGGAGGCCTGTACCTGGCTGTGACCC-GTTGGCAGGCATCACTGGAGTTGTCATCA                    0
C19   CTGAAGGAGGCCTGTACCTGGCTGTGACCCTGTTGGCAGGCATCACTGGAGTTGTCATCA                    -8
C20   CTGAAGGAGGCCTGTACCTGGC-------TGTTGGCAGGCATCACTGGAGTTGTCATCA                     -5
C21   CTGAAGGAGGCCTGTACCTGGCTGT-----TGTTGGCAGGCATCACTGGAGTTGTCATCA                    -1
C22   CTGAAGGAGGCCTGTACCTGGCTGTGACCC-GTTGGCAGGCATCACTGGAGTTGTCATCA                    -8
C23   CTGAAGGAGGCCTGTACCTGGCTGTGACCC--------GGCATCACTGGAGTTGTCATCA                    0
C24   CTGAAGGAGGCCTGTACCTGGCTGTGACCCTGTTGGCAGGCATCACTGGAGTTGTCATCA                    -6
C25   CTGAAGGAGGCCTGTACCTGGCTG------TGTTGGCAGGCATCACTGGAGTTGTCATCA
```

Supplemental Figure 7: Indel sequences recovered by shot gun cloning from LC-sgEx3- and LC-sgEx6-LV transduced cells.

Supplemental Table 1: Oligo nucleotides used in this study

**Oligo**		**Sequence (5′-3′)**
**Cloning of EGFP sgRNAs**		
BH001	g32DG1 s	CACCGGAGGAACTGTTCACCGGCG
BH002	g32DG1 as	AAACGTGAACAGTTCCTCGCCCTC
BH003	g32DG2 s	CACCGGAGGAACTGTTCACCGGCG
BH004	g32DG2 as	AAACCGCCGGTGAACAGTTCCTCC
BH037	g32DG1.4 s	CACCGGGGCGAGGAACTGTTTCAC
BH038	g32DG1.4 as	AAACGTGAAACAGTTCCTCGCCCC
BH039	g32DG1.7 s	CACCGAAGGGCGAGGAACTGTCAC
BH040	g32DG1.7 as	AAACGTGACAGTTCCTCGCCCTTC
BH041	g32DG2.3 s/ sgEGFP2.3 s	CACCGGGAACTGTTCACCGCGGCG
BH042	g32DG2.3 as/ sgEGFP2.3 as	AAACCGCCGCGGTGAACAGTTCCC
BH043	g32DG2.5 s	CACCGCGAGGAACTGTTCACGGCG
BH044	g32DG2.5 as	AAACCGCCGTGAACAGTTCCTCGC
**Cloning of CYBB sgRNAs**		
BH137	sgCYBB54 s	CACCGCAGCACTGGCACTGGCCCA
BH138	sgCYBB54 as	AAACTGGGCCAGTGCCAGTGCTGC
BH139	sgCYBB173 s	CACCGGTACCTGGCTGTGACCTGT
BH140	sgCYBB173 as	AAACACAGGTCACAGCCAGGTACC
BH141	sgCYBB45 s	CACCGTTTACACAAGAAAACGTCT
BH142	sgCYBB45 as	AAACAGACGTTTTCTTGTGTAAAC
BH143	sgCYBB124 s	CACCGACATCTATTTAATGTGTAA
BH144	sgCYBB124 as	AAACTTACACATTAAATAGATGTC
**Generation of EGFP mutant vectors**		
SFHR037	SBmGW s	ATGGTGTCCAAGGGCGAGGAACTGTTCACCGCGGCGTGGTGCCCATCCTG
SFHR038	SBmGW as	ATGGGCACCACGCCGCGGTGAACAGTTCCTCGCCCTTGGACACCATGGTT
SFHR046	SBmGW-K6 s	ATGGTGTCCAAGGGCGAGGAACTGTTCACCGTGGTGCCCATCCTG
SFHR047	SBmGW-K6 as	ATGGGCACCACGGTGAACAGTTCCTCGCCCTTGGACACCATGGTT
SFHR048	SBmGW-K16 s	ATGGTGTCCAAGGGCGAGGAACTGTTCACCGCGAGCGTGGTGCCCATCCTG
SFHR049	SBmGW-K16 as	ATGGGCACCACGCTCGCGGTGAACAGTTCCTCGCCCTTGGACACCATGGTT
SFHR050	SBmGW-K18 s	ATGGTGTCCAAGGGCGAGGAACTGTTCACCGCGGGCGTGGTGCCCATCCTG
SFHR051	SBmGW-K18 as	ATGGGCACCACGCCCGCGGTGAACAGTTCCTCGCCCTTGGACACCATGGTT
SFHR052	SBmGW-K29 s	ATGGTGTCCAAGGGCGAGGAACTGTTCACCGCGGTGCCCATCCTG
SFHR053	SBmGW-K29 as	ATGGGCACCGCGGTGAACAGTTCCTCGCCCTTGGACACCATGGTT
SFHR054	SBmGW-K33 s	ATGGTGTCCAAGGGCGAGGAACTGTTCACCGGCGCGGTGCCCATCCTG
SFHR055	SBmGW-K33 as	ATGGGCACCGCGCCGGTGAACAGTTCCTCGCCCTTGGACACCATGGGT
**Generation of CYBB mutant vectors**		
BH111	SB173CW s	GGGCGGCCTGTACCTGGCTGTGACCTGTTGGCCGGCATCACCGGCGTG
BH112	SB173CW as	CACGCCGGTGATGCCGGCCAACAGGTCACAGCCAGGTACAGGCCGCCC
BH113	SB54CW s	GCTGCTGGGCTCAGCACTGGCACTGGCCCAGGGCCCCTGCCGCCTGCCTGAA
BH114	SB54CW as	TTCAGGCAGGCGGCAGGGGCCCTGGGCCAGTGCCAGTGCTGAGCCCAGCAGC
BH121	SB124CW s	GCGCCATCCACACCATCGCACATCTATTTAATGTGTAATGGTGCGTGAACGCCCGGGTG
BH122	SB124CW as	CACCCGGGCGTTCACGCACCATTACACATTAAATAGATGTGCGATGGTGTGGATGGCGC
BH123	SB45CW s	CGACATCCCCCCCAAGTTCTTTTACACAAGAAAACGTCTTGGCAGCGCCCTGGCCCTGG
BH124	SB45CW as	CCAGGGCCAGGGCGCTGCCAAGACGTTTTCTTGTGTAAAAGAACTTGGGGGGGATGTCG
**Shotgun cloning**		
AR36	Shotg HPRT s	TCCCTTCATAGAGACAAGGAATG
AR163	Shotg HPRT as	CCTGGGTTCTACCCCAGCACAG
AR102	Shotg EGFP as	TCATGTGGTCGGGGTATCTG
SFHR20	Shotg EGFP as	CTTGTACAGCTCGTCCATGC
SFHR43	Shotg IRES s	TCTGTAGCGACCCTTTGCAG
BH234	Shotg CYBB as	AGGATCAGGCACAGGGTGAT
**Surveyor assay**		
BH229	Svyr45/54 s	TGGCTCTCCTCAAGCGTATT
BH230	Svyr45/54 as	CCATCTTGTGGAAGGTCAGG
BH231	Svyr173 s	CACCTGTTCAACGTGGAGTG
BH232	Svyr173 as	CTCGCACAGGTACAGGAACA
BH233	Svyr124 s	AACGTGTTCCTGTTCGTGTG
BH234	Svyr124 as	AGGATCAGGCACAGGGTGAT
SFHR 135	Svyr54_off1 s	ATTCCTTTCAACGCTAGCCTTC
SFHR 136	Svyr54_off1 as	GTACATTTGTTTGCCATCACCG
SFHR 137	Svyr54_off2 s	AAGCCATGGTCCAAAATCCTG
SFHR 138	Svyr54_off2 as	CCTGTTGTTCACAGCTCTATGT

SFHR141	Svyr54_off3 s	TGCAGGATCTGGTCCCTAATC
SFHR142	Svyr54_off3 as	GGAATCTCCCATCCTAGCCTAG
SFHR143	Svyr54_off4 s	CGGTCGTTGATTTCTGAGGAG
SFHR144	Svyr54_off5 as	ACAGAGCCTTGGTCTCATGA
SFHR145	Svyr173_off1 s	ACCCACTACGCACATATATATGC
SFHR146	Svyr173_off1 as	TAATGACCTGCAAACAAACCTAC
SFHR147	Svyr173_off2 s	CTCTGTGTTCAGCTCCAGC
SFHR148	Svyr173_off2 as	GTGATTCACAAACAAGGAGGTG
SFHR151	Svyr173_off3 s	CTTTGGCTCTGATGTTATATCTGTAG
SFHR152	Svyr173_off3 as	TCATCAGCATATCGTCTTGCC
SFHR153	Svyr173_off4 s	ACTTCTGCTTCAGGAATTGCC
SFHR154	Svyr173_off4 as	CTACTGGTGCCAGCAAAGAC

Mutation	DNA change	Protein Alteration	Accesion Number	sgRNA Sequence
SB*54*CW	c.159dupC	p.R54fs	A53X102	CAGCACTGGCACTGG**C**CCA**GGG**
SB*173*CW	c.517delC	p.L173fs	L173X188	GTACCTGGCTGTGACC**-**TGT**TGG**
SB*124*CW	c.370G>T	p.E124X	E124X	ACATCTATTTAATGTGT**T**AA**TGG**
SB*45*CW	c.134T>G	p.L45R	L45R	TTTACACAAGAAAAC**G**TCT**TGG**

Supplemental Table 2: Selected *CYBB* disease mutations and nucleotide sequences of the *CYBB* targeting sgRNAs.
